# The Use of Massive Sequencing to Detect Differences between Immature Embryos of MON810 and a Comparable Non-GM Maize Variety

**DOI:** 10.1371/journal.pone.0100895

**Published:** 2014-06-26

**Authors:** Jose Luis La Paz, Maria Pla, Emilio Centeno, Carlos M. Vicient, Pere Puigdomènech

**Affiliations:** 1 Department of Molecular Genetics, Center for Research in Agrigenomics, Consejo Superior de Investigaciones Científicas, CSIC-IRTA-UAB-UB, Barcelona, Spain; 2 Institute for Food and Agricultural Technology (INTEA), University of Girona, Campus Montilivi, EPS-I, Girona, Spain; University of New England, Australia

## Abstract

The insect resistant maize YieldGard MON810 was studied to assess the extent to which introduction of a transgene may putatively alter the expression of endogenous genes by comparison of various GM lines vs. their non-transgenic counterparts. To assess the extent to which introduction of a transgene may putatively alter the expression of endogenous genes, GM lines of the insect resistant maize YieldGard MON810 were compared with non-transgenic counterparts. For a more in-depth study, high-throughput deep sequencing together with microarrays were used to compare the transcriptomes of immature embryos of the MON810 variety DKC6575, with a *cryIA(b)* transgene, and its near-isogenic variety Tietar, grown under controlled environmental conditions. This technique also allows characterisation of the transgenic mRNAs produced. 3′UTR-anchored mRNA-seq produced 1,802,571 sequences from DKC6575 and 1,170,973 from Tietar, which mapped to 14,712 and 14,854 unigenes, respectively. Up to 32 reads from the transgenic embryos matched to the synthetic *cry1A(b)* sequence, similar to medium-abundant mRNAs. Gene expression analysis, using the R-bioconductor packages EdgeR and DEseq, revealed 140 differentially expressed genes mainly involved in carbohydrate metabolism, protein metabolism and chromatin organisation. Comparison of the expression of 30 selected genes in two additional MON810 and near-isogenic variety pairs showed that most of them were differentially expressed in the three pairs of varieties analysed. Analysis of functional annotation and the precise moment of expression of the differentially expressed genes and physiological data obtained suggest a slight but significant delay in seed and plant maturation of MON810 plants. However, these transcriptomic changes were not associated to undesirable changes in the global phenotype or plant behaviour. Moreover, while most expression changes in MON810 immature embryos were maintained in other transgenic varieties, some gene expression was found to be modulated by the genetic background in which the transgene was introduced through conventional breeding programs.

## Introduction

Genetically modified (GM) plants have been cultivated for 17 years, globally covering 160 million ha in 2011. Maize is the second major GM crop, occupying 32% of the global GM area and is the species with the highest number of events approved (65). The insect resistant maize YieldGard MON810 ranks as the second GM event to receive regulatory approval in most countries (23), after the herbicide tolerant soybean GTS-403–2 [1].

MON810 has a transgene cassette carrying an enhanced 35S transcription promoter derived from the cauliflower mosaic virus, the *hsp70* intron and a 5′ portion of a *Bacillus thuringiensis*-derived gene, *cryIA(b)*, encoding an insecticidal protein (∂-endotoxin) which controls lepidopteran pest insects such as the European corn borer. The same transgene cassette has been introgressed into several maize varieties. In the European Union, MON810 is the only GM event commercially cultured besides residual growth of a new biotech starch potato [1]. Characterisation of the transgene insertion site has demonstrated a 3′ truncation of the cassette resulting in lack of the termination sequence [2] and a complex recombination event associated with transgene integration. The sequence of the 5′ junction upstream from the 35S promoter shows 88% identity with the 22 kDa alpha zein gene [3] whereas the host 3′ flanking region corresponds to a gene putatively coding for the HECT E3 ubiquitin ligase, in the opposite orientation [4]. Read-through transcription of the MON810 transgene has been described as 3′ past the truncation site [4] generating a variety of polyadenylated transcripts of different lengths that extend 1 kb downstream of the truncation site [5]. As a result of a stop codon at position +7 downstream of this site, the transgenic protein includes two additional amino acids, which is compatible with the reported protein size [4], [5].

MON810 maize has been studied to assess to which extent the introduction of a transgene may putatively alter the expression of endogenous genes by comparison of various GM lines vs. their non-transgenic counterparts. Transcriptome profiles have been obtained from leaves and mature grains using microarray platforms [6], [7]. Even though microarrays contain probes corresponding to thousands of genes, a significant part of the maize genome, and particularly the transgene, is not covered. To further identify any unexpected effect of the transgene, we used massive mRNA sequencing to compare the transcriptomes of MON810 and conventional near-isogenic varieties, with particular emphasis on the host sequences flanking the transgene.

## Materials and Methods

### Ethics statement

No ethics approval was required. No animal trials were conducted. All experiments were performed on the laboratory of the consortium CSIC-IRTA-UAB-UB (CRAG). All plants were grown at CRAG greenhouses and all plant material residues and soil were destroyed after study was finished.

### Plant Material, growth conditions and data collection

Maize (*Zea mays*) seeds of commercial varieties of the YieldGard MON810 events DK6575 (Monsanto), PR33P67 (Pioneer Hi-Bred) and DKC6041-YG (Monsanto), and the near-isogenic varieties Tietar, PR33P66 and DKC6040 were obtained from the Spanish market. They are commercial hybrids that harbour the transgenic trait in hemizygous state. For each 454 RNA-seq experiment, twelve plants were grown to maturity in the greenhouse under controlled conditions (day 28±2°C, night 22±2°C, relative humidity 60±5%, 14/10 h light/dark, and controlled insect/fungal pathogens), and 100 embryos per plant (a total of 1200 embryos per variety) were collected 20 days after pollination//DAP. For *Zm-upl* gene expression analysis, the fourth leaf of plants at the seven leaf vegetative stage (FAO V7 developmental stage) was used. The MON810 homozygous line was obtained by auto-pollination of DKC6575 and absence of the wild type MON810 allele was assessed by PCR.

For measurements of embryo area (mm^2^), dry weight (mg) and embryo axis length (cm) a total of 60 embryos at 20 DAP and full maturity (>60 DAP) stages were excised from the mid-part of the cobs from twelve plants of the same three MON810-near isogenic variety pairs mentioned above. Total embryo area and axis length was calculated using high-resolution images (4928×3264 pixels) captured using a digital-reflex Nikon D-7000 camera. Contrasting background and conversing pixel-mm were performed using the ImageJ software [47]. For dry weight calculations, embryos were dried at 65°C oven over a week.

### ssc-DNA library construction and 454 mRNA sequencing

Total RNA was extracted from a total of 1,200 embryos per maize variety, DKC6575 and Tietar. They were ground in liquid N_2_, homogenised in TRIzol reagent (Invitrogen Life Technologies, Carlsbad CA, USA) and purified with the RNeasy Plant Mini kit (Qiagen, Hilden, Germany) according to the manufacturer's instructions. Polyadenylated mRNA was isolated from 15 µg of total RNA using the Oligotex mRNA kit (Qiagen). The cDNA first strand was synthesised from 5 µg poly-A+ mRNA with the Superscript II RT enzyme mix (Invitrogen Life Technologies) and a modified oligo(dT) primer which contained a *GsuI* restriction site (Table S1). The second-strand was synthesised with a combination of *E.coli* DNA polymerase I, *E.coli* DNA ligase and *E.coli* RNase H (Invitrogen Life Technologies), according to [8] and ds-cDNA was digested with *GsuI* and *MspI*. Top and bottom strands of A and B 454-adaptors (See Table S7 for adaptor sequences) were combined to a final concentration of 5 pM and allowed to anneal with a temperature increase of −1°C/min from 95°C to 60°C. Modified 454 A and B adaptors containing 2 bp overhangs (Table S1) were then directionally ligated to the digested ds-cDNA. After ligation, the 454-ds-cDNA libraries were bound to Dynabeads Streptavidin M-270 beads (Promega) by interaction with a Biotin tag included in the B-adaptor, according to the manufacturer's instructions. The libraries were immobilised on a magnabot-96 (Promega) and excess of adaptors and non-ligated products were washed twice with washing buffer (50 mM Tris-HCl, pH 8, 1 mM EDTA, 0.15 M LiCl). Finally, immobilised ds-cDNA molecules were denatured with 0.1 M NaOH. This step allows separation of the immobilised biotinylated strands from 5′A-cDNA-B 3′ strands that were finally eluted, pH neutralised and concentrated on a silica column using the Qiagen Minelute kit (Qiagen).

Single-strand 5′A-cDNA-B 3′ libraries were quality and quantity assessed using the Quant-iT Ribogreen fluorometric assay (Invitrogen Life Technologies) according to the manufacturer's instructions. Fragment length distribution was analysed by capillary micro-electrophoresis using an Agilent 2100 bioanalyzer. The two assays confirmed a total yield of >1×10^9^ molecules/µl in each library, with expected fragment lengths following a normal distribution with a mean of 150 bp, making them suitable for emulsion polymerase chain reaction (emPCR) titration assay.

Library titration, emPCR and sequencing were performed as per [9] in a 454 GS-20 instrument (454 Life Sciences, Roche, Branford, CT) using the FLX Titanium technology at the CRAG Massive Sequencing Service Facilities.

### 454 mRNA-seq data analysis

454 sequence output files were first quality trimmed and clipped for adaptor removal using the 454 GS-20 software. Valid sequences were filtered for the presence of modified poly-A+ tail and mapped against the maize genome using the maize genome release 5 database (http://www.maizesequence.org). Alignment to the maize reference genome release 5b.60 was using the gsMapper software (Roche). The alignment parameters were 90% of minimum identity between reads and the reference genome and 40 nucleotides as the minimum alignment region allowed, corresponding to the minimum read length given by the 454 analysis. A MySQL database was constructed with the assembled sequences including the MaizeGDB gene identifier, Unigene accession, Uniprot accession, ZmGi accession, gene evidence type, gene product description and GeneOntology annotations for each transcript-gene match. Read (sequence) frequency for each gene was established using the coverage value for each gene, which is the number of poly-A-read sequences that match unique transcript positions in the maize genome. Differential expression between libraries was assessed by DEseq and EdgeR statistic packages [10], [11] using a significance level *P* = 0.05 corrected with a false discovery rate (FDR) test [12] threshold of 10%. Functional classification of transcripts was based on GO terms and using the GORetriever software (http://www.agbase.msstate.edu/) and summarized using the GOSlimviewer software (http://www.agbase.msstate.edu/).

### Maize 44k Agilent Microarray hybridisations and data analysis

The Agilent 016047 maize 44K microarray (Agilent, Santa Clara, CA) was used to compare gene expression in 20 DAP embryos of MON810 and isogenic varieties. The chip has 42,032 probes to analyse approximately 35,727 *Zea mays* transcripts. Hybridisations were carried out at the CRAG Genomic Service facilities. Briefly, total RNA was extracted from pools of 50 maize embryos using the TRIZOL reagent (Invitrogen), and purified using the RNeasy kit (Qiagen). RNA integrity was checked by capillary micro-electrophoresis using the Bioanalyzer 2100 (Agilent), and samples with Integrity Ratios >9.0 were further used for cDNA synthesis and colour labelling. For cDNA synthesis, 1 µg total RNA was used. Amplification and Cy3/Cy5 labelling reaction was carried out by in vitro transcription using the Agilent Technologies Quick Amp Labeling Kit (Agilent) following the manufacturer's instructions. The cRNA concentration and labelling incorporation was checked using a Nanodrop spectrophotometer (Thermo Scientific, Wilmington, DE, USA), then fragmented to 50–100 nt and used for hybridisations at 65°C for 16–17 h. Finally, slides were washed and scanned using an Agilent DNA microarray Scanner (Agilent). Data extraction, including background adjustment, experimental replicate normalisation, and intensity normalisation and differential expression statistical test was performed using the Robin software [13]. Quality control consisted of background subtraction and intensity signal normalisation using the Prin Tip Loess method [14]. The threshold for differentially expressed genes was p<0,05, adjusted with a FDR threshold of 10%, calculated as described previously. Functional classification of transcripts was based on GO terms and using the GORetriever software (http://www.agbase.msstate.edu/) and summarised using the GOSlimviewer software (http://www.agbase.msstate.edu/).

### Data validation by quantitative real-time PCR

The expression of 14 differentially regulated sequences was assayed by reverse transcription coupled to real-time PCR (RT-qPCR) to validate the results of the 454 sequencing and microarray, and for further expression analyses in different maize tissues and varieties. Total RNA was extracted as mentioned previously from 100 mg of frozen tissue. RNA was treated with DNAse I to fully eliminate DNA prior to reverse transcription. Purified RNA was quantified by UV absorption using a Nanodrop 1000 spectrophotometer (Thermo Scientific, Wilmington, DE), and the absence of remaining DNA was demonstrated by RT-PCR of RNA samples. First strand cDNA synthesis and RT-qPCR amplification were carried out simultaneously using the SuperScript III Platinum SYBR Green One-Step qPCR Kit (Invitrogen Life Technologies). Each reaction was in a 25 µl final volume containing 0.5 µg total RNA and 0.2 µM of each target-specific primer. The oligonucleotides, shown in Table S1, were purchased from Eurofins-MWG Biotech AG (Germany). RT-qPCR reactions were carried out in a Lightcycler LC480 device (Roche) with the following run program: 10 min denaturation at 95°C, 45 repeats of 15 s at 95°C and 30 s at 60°C, and a melting curve analysis (60–95°C with a heating rate of 0.5°C/s). The specificity of the PCR was demonstrated by the melting curve analysis, which consistently gave single peaks. All reactions were in triplicate. Linearity (R^2^) and efficiency (E = 10^[−1/slope]^)[15] of every reaction were within the accepted values. Maize housekeeping genes encoding Actin-1, Tubulin-α4, Tubulin-β5, GAPDC2, GAPDC3, Cyclophilin and Ubiquitin were initially assessed and *cyclophilin* was the most stable in our samples (NORMfinder M<0.50). Relative gene expression was calculated using the 2^−ΔΔCt^ method. The geNORM software was used for data normalisation and statistical analyses (*t*-test).

### ABA quantification

ABA levels were quantified in three different MON810-near isogenic variety pairs (DKC6575-Tietar; PR33P67-PR33P66; DKC6041YG-DKC6040) using the Phytodetek ABA ELISA kit (Agdia, Inc, Elkhart, IN) according to the manufacturer's instructions. ABA was determined in 20 DAP embryos, 50 DAP embryos and mature-dry embryos in triplicate pools of 100 embryos per variety and developmental stage. Statistical significance was assessed by one-way ANOVA with Tukey's b post-test, with significance level set at 0.05.

### *In vitro* germination monitoring assay

Seeds from MON810 and near-isogenic counterparts (var. DKC6575 and Tietar) were first sterilized in 70% ethanol and 1% NaOCl, 0.01% Tween-20, then 250 seeds per variety were grown in 30 cm high jars on MP media (4.4 g/L MS including vitamins, 50 g/L sucrose) in a growth chamber under controlled conditions of 29°C and a 12 h light, 12 h darkness photoperiod. Radicle and plumule length were measured over the following nine days, and seeds were classified according to the scale: 0 mm (no emerging radical or plumule), <10 mm, 10-30 mm, and >30 mm.

## Results

### Characterisation of expression profiles in MON810 and near-isogenic maize embryos

The Roche 454 GS-FLX (Titanium) pyrosequencing technology [9] was used to analyse cDNA samples from 20 days after pollination (DAP) embryos of the commercial MON810 transgenic maize variety DKC6575 and its near isogenic conventional variety Tietar. Two ss-cDNA libraries were synthesised from polyadenylated mRNAs with a modified protocol [16] that reduces the poly-(A+) tails to 3 adenines (Figure S1). The two ss-cDNA libraries yielded a total of 2.9 M raw reads (submitted to the NCBI Short Read Archive; http://trace.ncbi.nlm.nih.gov/Traces/sra/sra.cgi; Acc number EMBL: E-MTAB-1555). After filtering and clipping, this gave more than 400k poly-A 3′UTR anchored reads for each variety (Table 1). We selected 3′UTR anchored reads to reduce the possibility of assigning a single read to more than one gene in the read-to-genome mapping analysis.

**Table 1:** 454 3′UTR-mRNAseq run statistics and bioinformatics data processing.

	DKC6575 (MON810)	Tietar (near-isogenic)
**454 Run statistics**		
Raw reads	1082571	1170973
Total bases	90278915	75157511
TCAG key passed reads	1067685	1158771
Read length average (nt)	174	139
Valid reads after quality filtering (dot, mixed, primer)	570465	629721
**Data analysis and alignment parameters**		
Total mapped reads	531217	597227
3′UTR poly-A key passed reads	457181	449018
Read length average (nt)	197	167
Total bases	40417894	47572825
Non redundant reads matching unique transcripts	173541	206072
Avg. Alignment length	154	161

Selected reads were aligned with the B73 maize genome version 5b.60 [17], available at the Maize Genome Sequencing Project web (www.maizesequence.org), considering positive those reads showing at least 90% identity with the reference, with a minimum alignment region of 40 nucleotides, which corresponds to the minimum read length given by the 454 software. Unique transcripts were represented by 1 to 8,000 reads. Uniquely mapped reads corresponded to 14,712 and 14,854 unigenes in the transgenic and near-isogenic varieties, respectively (Table 1). Analysis of the identified unigenes was using GO-retriever and GO-slimviewer, which classified them in 55 high level biological process and molecular activity GO categories (Table 2). Using the AgriGO tool [18] for a singular enrichment analysis (SEA) for the GO categories of biological process and molecular function there were no significant GO categories overrepresented comparing a total of 9695 GO categories between DKC6575 and Tietar (p-value <0.05; FDR 10%).

**Table 2:** Clustering of genes expressed in DKC6575 and Tietar 20 DAP embryos, as assessed by mRNA-seq and Agilent 44k microarray hybridization, on the basis of the biological process and molecular activity GeneOntology high-level categories.

	454 mRNA-seq				Agilent microarray			
	DKC6575 (MON810)		Tietar (near-isogenic)		DKC6575 (MON810)		Tietar (near-isogenic)	
**Biological process**	**No. genes**	**%**	**No. genes**	**%**	**No. genes**	**%**	**No. genes**	**%**
**GO Category**								
Metabolic process	5442	23.41	5507	23.34	3071	17.30	3140	17.40
Cellular process	5076	21.83	5152	21.83	3432	19.40	3398	18.90
Biosynthetic process	2063	8.87	2136	9.05	995	5.60	1064	5.90
Biological process	1963	8.44	1999	8.47	2640	14.90	2704	15.00
Nucleoside and nucleotide metabolism	1649	7.47	1726	7.61	858	4.80	793	4.40
Transport	1321	5.68	1323	5.61	732	4.10	708	3.90
Transcription	1265	5.44	1345	5.70	542	3.01	547	2.99
Protein modification process	653	2.81	650	2.75	476	2.70	438	2.40
Translation	568	2.44	572	2.42	202	1.10	241	1.30
Carbohydrate metabolic process	510	2.19	479	2.03	315	1.80	349	1.90
Protein metabolic process	507	2.18	512	2.17	343	1.90	342	1.90
Catabolic process	394	1.69	386	1.64	311	1.80	302	1.80
Lipid metabolic process	321	1.38	316	1.34	238	1.30	245	1.40
Signal transduction	309	1.33	294	1.25	277	1.60	272	1.50
Cellular component organization	293	1.26	277	1.17	395	2.20	374	2.10
Response to stress	233	1.00	250	1.06	696	3.90	777	4.30
DNA metabolic process	166	0.71	180	0.76	218	1.20	189	1.10
Generation of precursors and energy	129	0.55	118	0.50	83	0.50	100	0.60
Cellular homeostasis	97	0.42	94	0.40	51	0.30	55	0.30
Response to endogenous stimulus	74	0.32	73	0.31	244	1.40	266	1.50
Cell cycle	43	0.18	37	0.16	98	0.60	88	0.50
Response to abiotic stimulus	34	0.15	39	0.17	471	2.70	531	3.00
Secondary metabolic process	19	0.08	24	0.10	24	0.10	23	0.10
Cell communication	10	0.04	9	0.04	46	0.30	49	0.30
Response to external stimulus	8	0.03	11	0.05	44	0.20	53	0.30
Response to biotic stimulus	7	0.03	7	0.03	166	0.90	189	1.10
Regulation of gene expression, epigenetics	7	0.03	7	0.03	38	0.20	38	0.20
Embryo development	2	0.01	2	0.01	75	0.40	75	0.40
Cell differentiation	1	<0.01	1	<0.01	83	0.50	72	0.40
**Molecular Activity**	**No. genes**	**%**	**No. genes**	**%**	**No. genes**	**%**	**No. genes**	**%**
**GO category**								
Binding	3081	17.61	3139	17.82	2805	15.59	2769	15.39
Catalytic activity	2808	16.05	2835	16.09	2383	13.24	2688	14.93
Transferase activity	2367	13.53	2347	13.32	2924	16.25	2032	16.85
Nucleotide binding	2088	11.93	2080	11.81	2458	13.66	2230	12.39
Hydrolase activity	1837	10.50	1887	10.71	2212	12.29	2051	11.40
Kinase activity	1163	6.65	1120	6.36	1229	6.83	1212	6.74
DNA binding	864	4.94	907	5.15	805	4.47	793	4.41
Nucleic acid binding	493	2.82	489	2.78	488	2.71	441	2.45
Transporter activity	473	2.70	471	2.67	578	3.21	556	3.09
Structural molecule activity	405	2.31	401	2.28	535	2.97	550	3.06
Protein binding	388	2.22	402	2.28	535	2.97	550	3.06
Molecular function	297	1.70	304	1.73	206	1.15	248	1.38
RNA binding	277	1.58	275	1.56	307	1.71	316	1.76
Sequence-specific DNA binding activity	230	1.31	244	1.39	207	1.15	224	1.25
Transcription regulator activity	158	0.90	155	0.88	99	0.55	97	0.54
Enzyme regulator activity	126	0.72	126	0.72	112	0.63	105	0.59
Signal transducer activity	83	0.47	78	0.44	99	0.55	97	0.54
Translation factor activity	83	0.47	81	0.46	65	0.37	89	0.50
Receptor activity	74	0.42	72	0.41	74	0.41	90	0.50
Nuclease activity	61	0.35	70	0.40	108	0.60	81	0.45
Carbohydrate activity	59	0.34	48	0.27	63	0.35	53	0.30
Lipid binding	44	0.25	41	0.23	69	0.38	67	0.38
Motor activity	28	0.16	32	0.18	46	0.26	27	0.15
Chromatin binding	5	0.03	5	0.03	7	0.04	11	0.06
Receptor binding	5	0.03	5	0.03	1	0.01	1	0.01
Oxygen binding	3	0.02	3	0.02	1	0.01	1	0.01

We analysed MON810 and conventional embryo transcriptomes using hybridisation to the Agilent 016047 maize 44K microarray. The 42,034 reporters included in the array were filtered to discard inconclusive (6,485) and ambiguous (2,608) reporters, according to the annotation from aligning all reporter probes with the maize B73 RefGen v2 “Working Gene Set” [19] (Submitted to the EBI ArrayExpress databank Acc number EBI-EMBL: E-MTAB-1558).

A total of 19,234 and 19,342 expressed unigenes were identified in MON810 and Tietar 20 DAP embryos, respectively. A GO enrichment analysis was performed as previously, and genes expressed in MON810 and isogenic varieties were found to be distributed in virtually the same GO categories (p-value <0.05; FDR 10%; Table S1). Only poorly represented GO categories gathered notably higher numbers of unigenes identified in microarrays hybridization than mRNA-seq experiments (using p-value <0.05 and FDR 10%). These differences are most probably a consequence of the identity of the reporters included in the Agilent microarray.

### Identification of genes with differential expression in DKC6575 and Tietar maize embryos

In analysing mRNA-seq results we used coverage as an indicator of gene transcription. Coverage (i.e. number of reads mapping at the same genomic position) was calculated for each genomic region that contained unique mapping reads. This allowed the calculation of the percentage of mRNA molecules that corresponded to a given gene, assuming that one read is equivalent to one mRNA molecule. Genes showing differential expression in DKC6575 and Tietar embryos were identified using the R bioconductor packages DEseq [10] and EdgeR [11]. A total of 5022 unigenes were included in the analysis. Unigenes with less than five reads were excluded from statistical analyses [20]. DEseq and EdgeR packages detected 2.8% (148 and 145 respectively) of genes included in the analysis as differentially expressed between MON810 and near-isogenic variety embryos (log2-fold-change above 1 or below -1; p-value <0,05; adjusted with BH correction FDR 10% test [12]). Up to 140 (95%) differentially expressed sequences were commonly detected using the two alternative packages. Among genes identified using the DEseq package, 55 were up-regulated and 93 were down-regulated. The EdgeR package issued 50 genes up-regulated in transgenic embryos and 95 genes down-regulated in this variety (Table S2 and S3). Relative expression versus read count MA-plots of all genes are shown in Figure S2A and S2B.

Sequences exhibiting differential expression in MON810 and conventional embryos were identified in parallel by analysing our microarray hybridization results using the ROBIN software [13]. Up to 122 probes had different intensities upon hybridization with DKC6575 and Tietar cDNA (log2-fold-change above 1 or below -1; p-value <0,05; adjusted with BH correction FDR 10% test [12]), which represented 0.63% of the sequences giving positive signal. Forty-eight genes were up-regulated and 74 down-regulated in MON810 (Table S4). Figure S2C shows the relative expression (log2-fold-change) of all probes in an MA-plot.

**Figure 1:**
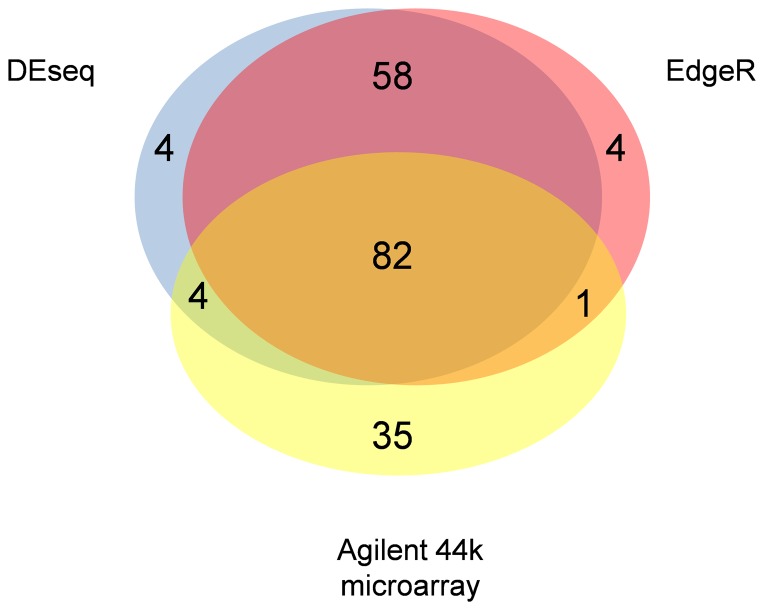
Venn diagram showing differentially expressed genes that were identified by mRNAseq coupled to the DEseq (blue) and EdgeR (red) packages; and Agilent 44k microarray hybridization (yellow).

Most genes identified as differentially expressed in DKC6575 and Tietar 20 DAP embryos appeared upon mRNA-seq analysis using both DEseq and EdgeR; and upon microarray hybridization (Figure 1). Table S1 shows their identification number, description and relative expression levels. There were 87 sequences which differential expression was commonly detected by mRNA-seq and microarray hybridization. Additionally, 58 genes were identified as differentially expressed using mRNA-seq coupled to DEseq and EdgeR packages but not by microarray hybridization. Upon searching the Agilent 44K maize microarray probeset we observed that they were not represented in the microarray. Thus, they were included in the candidate differentially expressed gene list. Similarly, the 35 sequences showing differential expression exclusively through microarray hybridization were searched among the mRNA-seq results. There were reads representing all 35 unigenes in the two sscDNA libraries, but read count were similar in DKC6575 and Tietar samples and were discarded from subsequent analyses.

### Characterization of genes showing differential expression in DKC6575 and Tietar 20 DAP embryos

A parametric analysis of gene set enrichment was performed to determine whether genes differentially expressed in DKC6575 and Tietar (Table S1) preferentially clustered into specific GO categories, using the AgriGO tool. Although a large proportion of differentially expressed genes are still uncharacterized and there is no GO reference available, five high-level GO categories were significantly overrepresented in the biological process domain, i.e. carbohydrate metabolism, chromatin organization-DNA packaging, protein modification, ribosome constituent and protein biosynthesis-branched amino-acid synthesis (Table 3). These results suggest that some genes regulated in DKC6575 and Tietar do preferentially have these specific biological functions. We selected 30 differentially expressed genes that clustered in overrepresented GO categories (15 up- and 15-downregulated) and further confirmed their differential expression in DKC6575 and Tietar embryos using real-time qPCR (Table 4). This alternative approach additionally validated mRNA-seq and microarray hybridization data (Table S5).

**Table 3:** Singular enrichment analysis (SEA) of biological process GO categories clustering candidate genes differentially expressed in MON810 and near-isogenic embryos.

GO terms	Description	Genes/total DEG^1^	Genes BG/Ref^2^	*p-value*	Adj. *p-value* FDR 10%
GO:0005975	Carbohydrate metabolism	21/140	1828/39203	0.0001	0.015
GO:0006325 GO:0006323	Chromatin organization DNA packaging	7/140	323/39203	0.0002	0.015
GO:0036211	Protein modification	19/140	320/39203	0.0002	0.005
GO:0003735	Constituent of ribosome	9/140	1390/39203	0.0014	0.023
GO:0006412 GO:0009082	Protein biosynthesis Branched-amino acid synthesis	9/140	1871/39203	0.0014	0.048

1 DEG: Differentially expressed genes.

2 BG/Ref: Genes in a given category/total genes in database with GO terms for that specie.

**Table 4:** RT-qPCR mRNA expression levels of 30 genes showing differential expression in DKC6575 and Tietar 20 DAP embryos, in immature embryos of the three MON810 and near-isogenic variety pairs: DKC6575 and Tietar, PR33P67 and PR33P66, and DKC6041-YG and DKC6040.

	Log2 Fold change			
**Gene ID**	**6575/Tietar**	**P67/P66**	**6041YG/6040**	**Description**
GRMZM2G466833	2.3722	2.292	2.125	Malate dehydrogenase
GRMZM2G027862	2.3084	2.019	1.221	Cellulose synthase 1
GRMZM2G134251	1.4256	1.328	1.447	Putative ubiquitin family protein isoform 1
GRMZM2G028286	1.3481	1.383	1.599	Proteasome activator subunit 4 putative
GRMZM2G447795	2.1221	1.452	1.113	E3-Ubiquitin ligase related
GRMZM2G013811	1.8321	1.465	1.125	4-alpha-glucanotransferase putative
GRMZM2G172369	1.9822	1.613	1.275	Mannose binding-protein
GRMZM2G028955	1.4820	1.135	1.034	Histone H2A6
GRMZM2G421279	1.4178	1.249	1.124	Histone H4C14
GRMZM2G072855	1.5128	1.284	1.218	Histone H4C7
GRMZM2G181153	1.3904	1.061	1.143	Histone H4C13
GRMZM2G151826	1.3105	0.965*	1.035	Histone H2A2
GRMZM2G093325	1.8723	0.927*	0.845*	Early response to dehydration-15
GRMZM2G015605	1.1144	0.669*	0.864*	Dehydration protein putative
GRMZM2G040095	1.1216	0.868*	0.929*	Lipoxygenase
GRMZM2G097229	−1.1430	−0.998*	−0.999*	Expansin B4
GRMZM2G102230	−1.2241	−1.010	−1.070	60S ribosomal protein L23
GRMZM2G327564	−1.6322	−1.088	−1.462	60S ribosomal protein L26-1
GRMZM2G104632	−1.2846	−1.090	−0.921*	Cellulase
GRMZM2G118003	−1.5287	−1.100	−0.930*	Pectinesterase
GRMZM2G167637	−1.6996	−1.344	−1.175	Endo-Beta-Mannanase
GRMZM2G140201	−2.0000	−1.525	−1.256	leucyl-tRNA synthetase
GRMZM2G174757	−2.1423	−1.177	−0.971*	Putative tryptophan synthase alpha
GRMZM2G100120	−2.5356	−1.585	−1.560	Hexose transporter
GRMZM2G071333	−2.4030	−1.832	−1.877	Thiamine thiazole synthase 1
GRMZM2G046191	−1.9695	−1.705	−1.999	Acetolactate synthase
GRMZM2G083173	−1.7884	−1.560	−2.022	Putative CAD
GRMZM2G018375	−1.9265	−1.968	−2.268	Xyloglucan glycosyltransferase 10
GRMZM2G407044	−1.8206	−1.926	−1.845	Xylanase inhibitor protein 1
GRMZM2G179981	−2.7549	−1.641	−1.245	Beta-hexosaminidase

Highlighted in grey are log2 fold change values not fulfilling the +1 to -1 cut-off value.

*Values out of log2 fold-change threshold of +1 to −1.

The same experimental approach was used to assess to what extent the observed transcriptome differences in DKC6575 and Tietar embryos were conserved in other MON810 and near-isogenic variety pairs. mRNA levels of the 30 selected genes were quantified in 20 DAP embryos extracted from plants of two additional variety pairs grown under the same conditions: PR33P67 (MON810) and PR33P66 (near-isogenic), bred by Pioneer Hi-Bred; and DKC6041-YG (MON810) and DKC6040 (near-isogenic), bred by Monsanto. Up to 22 analysed genes (73%) were differentially expressed in all three pairs of MON810 and near-isogenic conventional varieties, five genes were similarly expressed in PR33P67/PR33P66 and seven in DKC6041YG/DKC6040 (Table 4). Note that these genes have similar log2 fold-change values in the +0.845 to -0.971 range, i.e. close to the established threshold.

These results indicate that the presence and/or expression of the truncated *cry1A(b)* transgene in the MON810 event may entail some transcriptomic changes in immature embryos, although the genetic background in which the transgene has been introduced modulates these changes in a specific manner.

### Characterisation of *cryIA(b)* transgene transcripts and those of the flanking gene *zm-upl*

**Figure 2:**
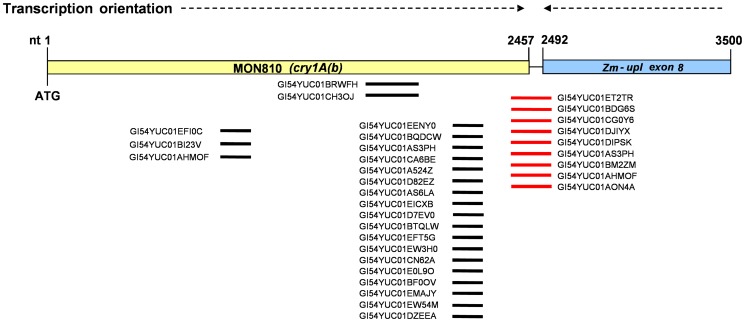
mRNA-seq reads matching the *cry1A(b)* transgene coding sequence, mapped over a schematic representation of the 3' region of the MON810 transgene locus. The inserted *cry1A(b)* transgene, in antisense orientation, truncates exon 8 of the HECT-E3 ubiquitin ligase (z*m-upl*) host gene. Polyadenylated reads are shown in red.

The mRNA-seq sequences were screened against the complete *cryIA(b)* coding region to assess the expression of the *cryIA(b)* transgene. None of the reads obtained from the conventional embryo samples contained any sequence homologous to *cryIA(b)*, but 32 reads from the transgenic embryos were similar to *cryIA(b)* (Table S6; Figure 2). Nine of the sequences corresponding to the *cryIA(b)* transgene extended downstream from the described *cryIA(b)* sequence and terminated in the adjacent 3′ genomic region flanking the transgene, which is similar to an antisense gene coding for a HECT type E3 ubiquitin ligase (*zm-upl*) (Figure 2 in red). This confirms the production of chimeric poly-A+ transcripts in MON810, encompassing *cryIA(b)* linked to antisense *zm-upl* sequences. Conversely, no antisense *cryIA(b)* reads were detected, which indicated the absence of chimeric transcripts encompassing *zm-ulp* and antisense *cryIA(b)*.

**Figure 3:**
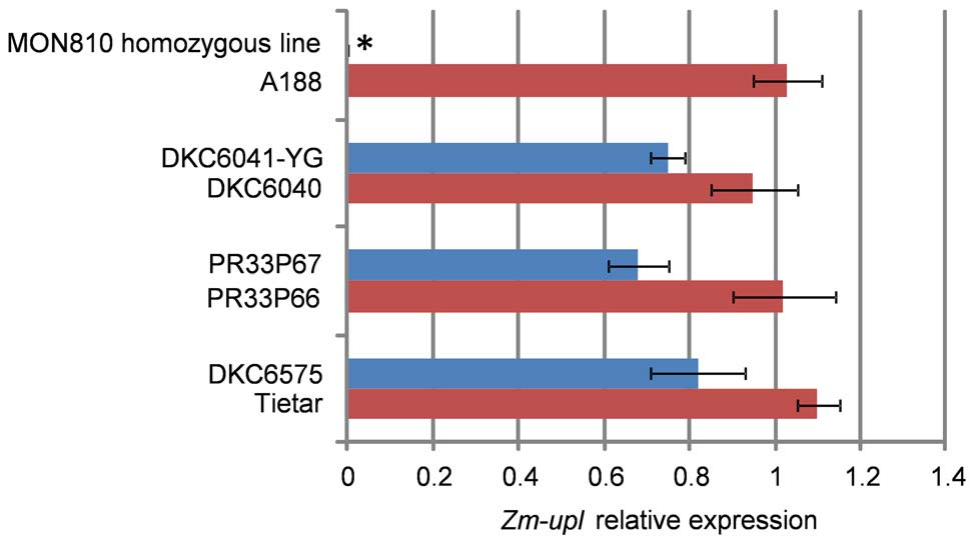
Relative expression of *zm-upl* in three MON810-near isogenic variety pairs (DKC6041YG/DKC6040; PR33P67/PR33P66; DKC6575/Tietar), the conventional non-agronomic line A188 and a non-commercial MON810 homozygous line. Means and SD are shown. Blue bars, transgenic varieties; red bars conventional varieties. The asterisk indicates statistically different mRNA values (one-way ANOVA, Tukey's post-test α<0.05).

DKC6575 (MON810) plants are hemizygous for the transgene, so they contain an intact copy of the E3 ubiquitin-ligase gene (in addition to the truncated copy 3′ flanking the transgene). Detection of chimeric transcripts containing antisense *zm-upl* sequences in these plants makes silencing of *zm-upl* possible. DKC6575 and Tietar mRNA-seq datasets were blasted against a 400 bp sequence corresponding to exon 8 of *zm-upl*, giving seven and six reads, respectively. This seemed to discard an inhibitory effect of chimeric *cryIA(b)* transcripts over *zm-upl* expression. *Zm-upl* expression was subsequently assessed by RT-qPCR in V2 stage leaves of a number of MON810 and the corresponding near-isogenic conventional varieties (Figure 3). Similar zm-upl mRNA levels were found in DKC6575 and Tietar, PR33P67 and PR33P66, and DKC6041-YG and DKC6040 (t-test P>0.05). The non-commercial variety A188, commonly used to transform maize, was used as an independent conventional control for comparison and had similar *zm-upl* expression as MON810 varieties (one-way ANOVA, Tukey's b post-test α>0.05), indicating the analysed commercial MON810 varieties had *zm-upl* expression values in the range of conventional ones. Finally, we analysed the expression of *zm-upl* expression in leaf samples taken from MON810 homozygous lines obtained by self-crossing DKC6575 in the greenhouse. *Zm-upl* expression was undetectable in the homozygous line (Figure 3).

### Physiological and morphological analysis of 20 DAP embryos of MON810 and near-isogenic varieties

To test if there were differences in the embryo physiology and/or morphology that could explain the observed transcriptomic differences, we collected some data relevant to embryo development at the 20DAP stage. The hormone ABA controls acquisition of desiccation tolerance, one of the key processes during the late embryogenesis phase. We measured ABA contents in embryos of three MON810-near isogenic variety pairs [Figure S3]. Twenty DAP embryos of all three MON810 commercial varieties (DKC6575, PR33P67 and DKC6041-YG) had similar ABA contents to their corresponding near-isogenic counterparts (Tietar, PR33P66 and DKC6040, respectively) (Figure S3). However, there were statistically significant differences in the ABA levels of 50 DAP embryos, MON810 embryos displaying lower levels than their near-isogenic conventional counterparts. These differences were slightly reduced in fully mature embryos (Figure S3). We then checked whether ABA differences could have an effect on the germination process and initial plant development. In an in-vitro germination assay we compared DKC6575 and Tietar by measuring the radicle and plumule emergence and growth during nine days upon imbibition. Germination of mature DKC6575 seeds was quicker than that of Tietar (Figure S4). Radicle emergence was visible in MON810 one day before than in conventional seeds; and 3 days after imbibition about half transgenic seeds had 10–30 mm long radicles whereas most conventional ones were less than 10 mm long. Differences in plumule emergence and growth were clearly visible at days 7 and 9 after imbibition, most MON810 but just half conventional ones reaching 30 mm.

**Figure 4:**
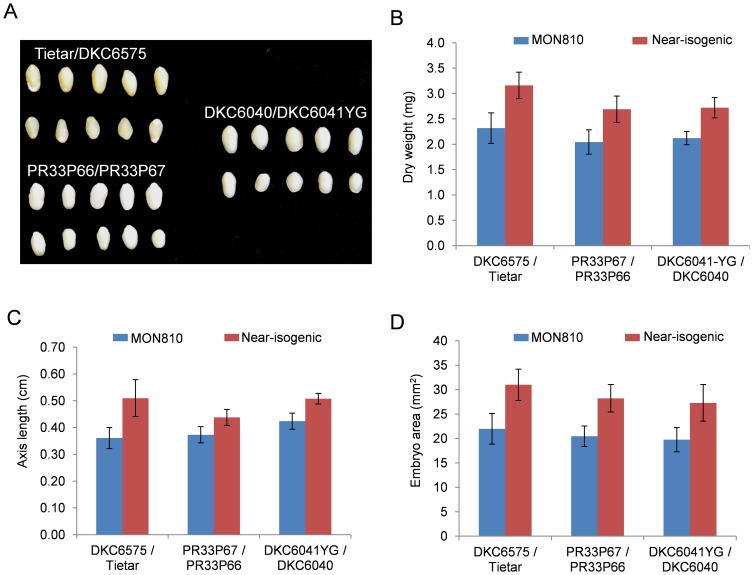
Morphological data collected from 20 DAP embryos of MON810 –near isogenic variety pairs DKC6575/Tietar; PR33P67/PR33P66 and DKC6041YG/DKC6040. **A**) embryo phenotype at developmental stage of 20 DAP. Dry weight of embryos (**B**), embryo axis length (**C**), and embryo area (**D**) physical parameters measured in 50 mid-ear embryos. Means and SD are shown. Blue bars, transgenic varieties; red bars conventional varieties.

We finally analysed some morphological parameters in 20DAP embryos of the same variety pairs. There were differences between MON810 embryos and the corresponding near-isogenic ones in terms of dry weight (Figure 4B), axis length (Figure 4C) and total area (Figure 4D), GM embryos being slightly smaller than the conventional ones (Figure 4A). These differences might indicate changes in the developmental timing between MON810 and near-isogenic embryos. However, there were no statistical differences in these morphological parameters between GM and near-isogenic fully mature embryos, indicating that MON810 and conventional varieties are similar at harvest (Table S8).

## Discussion

The GM maize event MON810 is extensively grown [1]. Many different varieties are commercialised that are genetically diverse but contain the same transgene inserted at the same chromosomal location. The aim of the present study was to assess possible consistent differences between the transcriptome of immature embryos of commercial MON810 and near-isogenic non-GM varieties, with special emphasis on the transgene and flanking sequences. The use of high-throughput massive sequencing should provide deep and unbiased transcriptomic information [21], [22], [23], allowing identification of small differences between transgenic and conventional transcriptomes and thus, detection of unintended effects of the transformation event [24]. We did not detect strong changes in the gene sets expressed in the MON810 variety DKC6575 and its near-isogenic counterpart; and genes expressed in the GM and/or the non-GM variety did not cluster in a specific, overrepresented GO categories. Even so, up to 140 genes were expressed at different levels in DKC6575 and Tietar immature embryos (Table S1). This corresponded to about 0.95% and 0.94% maize genes detected as expressed both in DKC6575 and Tietar 20 DAP embryos. This is in accordance with previous reports on transcriptome comparisons of various GM *versus* no-GM plants [24]; [25].

mRNA-seq results were further validated by comparing DKC6575 and Tietar embryo transcriptomic profiles using a microarray hybridisation approach with the Agilent platform, questioning around 33,000 genes. A total of 122 probes (i.e. 0.8% maize genes in the microarray and 0.6% genes detected to be expressed both in DKC6575 and Tietar 20 DAP embryos) had significant hybridisation differences in GM and conventional samples (Table S4). These values are similar to those obtained by mRNA-seq and also to those reported in MON810 versus near-isogenic leaf tissue [7], [25] and mature grains grown in agricultural fields [26], [27], [28]. Although the general numbers of regulated sequences are similar by using mRNA-seq (140) and microarray hybridization (122), just 82 sequences (about 60%) were identified through the two technical approaches. Fifty-eight additional sequences were only identified by mRNAseq and 35 were uniquely detected using microarray hybridization. This reflects the differences inherent in each technology [29], [30], [31]. Unigenes with less than five reads were discarded from mRNA-seq analysis carried out to identify differentially expressed genes, thus genes with low expression levels were not investigated using this approach. Similarly all 58 gene transcripts showing differential expressed in GM and conventional embryos as determined by mRNA-seq were not included or annotated in the microarray probeset, thus they were not investigated in the microarray hybridization experiment. Our double experimental approach, coupled to RT-qPCR expression analysis of 30 sequences, allowed concluding with high confidence that 140 candidate genes listed in Table 2 were differentially expressed in DKC6575 and Tietar 20 DAP embryos.

### Genes differentially expressed in MON810 variety DKC6575

**Figure 5:**
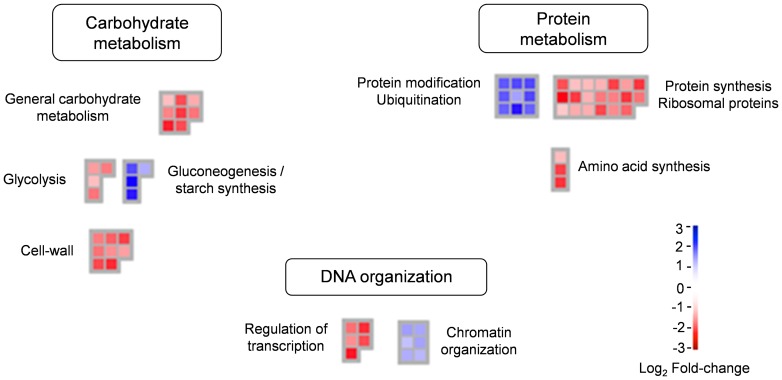
Mapman based representation of biological function categories enriched in genes differentially expressed in MON810 versus near-isogenic immature embryos. Each gene is represented as a box; red boxes indicate genes down-regulated in the MON810 variety and blue boxes correspond to genes up-regulated in DKC6575 in a log2 scale of +3 to -3.

We next investigated whether the changes identified in MON810 and near-isogenic immature embryos resulted in an effect on specific biological processes. We used mapman software [32] to assess the biological processes associated to every differentially expressed gene and to cluster those involved in similar processes (Figure 5). Fifteen percent of differential genes were involved in carbohydrate metabolism, most specifically in cell-wall synthesis or modification, e.g. *cellulose-synthase (ces-A)*, *xyloglucan endotransglycosylase (xth)* and the group of genes belonging to the B-glycosylase enzymes. These were all down-regulated in the transgenic embryos. Cell-wall enzymes such as the *xth*, or the *xylanase inhibitor (xipI)* have been described to display high expression levels in the embryo, specifically during early seed development [33], [34], and their transcriptional control is strongly repressed by drought stress and the hormone abcissic acid (ABA) [35], [36]. Other genes regulated by ABA were down-regulated as well in MON810 20 DAP embryos, e.g. those encoding a cellulase (GRMZM2G118003), an expansin B4 (GRMZM2G097229) and the drought-induced protein lti6b (GRMZM2G093325). These results seemed to indicate that DKC6575 and Tietar 20 DAP embryos had different contents of ABA. However, we experimentally showed that 20 DAP MON810 embryos of three different commercial varieties DKC6575, PR33P67 and DKC6041-YG had similar ABA contents to their near-isogenic counterparts Tietar, PR33P66 and DKC6040, respectively (Figure S3). Thus, the observed differences in cell wall genes could not be attributed to differences in ABA content, at least at the 20 DAP embryogenesis stage. Upon grain development, embryos of the same MON810 varieties had lower ABA contents than their conventional counterparts, as assessed using 50 DAP and physiologically mature embryos.

Four additional genes, related to carbohydrate metabolism and specifically clustering in the glycolysis pathway, were repressed in MON810 embryos (Figure 5). Some maize glycolysis genes have been reported to be up-regulated in the embryo at early (15DAP) and later (27DAP) developmental stages and to have low mRNA levels during intermediate stages (21DAP) [37].

Up to one forth genes differentially expressed in DKC6575 and Tietar 20 DAP embryos clustered in overrepresented categories related to DNA organization and protein metabolism. In particular, genes in the chromatin organization and protein modification (ubiquitination) categories were up-regulated in MON810, whereas those in the protein and amino acid biosynthesis and synthesis of ribosomal constituents, as well as those in the transcription regulation clusters, were down-regulated in transgenic samples. This seems to indicate that MON810 embryos would display lower protein biosynthesis processes and increased chromatin restructuration and protein ubiquitination (Figure 5). Young maize embryos are known to highly express a number of genes related to protein degradation [38], [39]; and embryos at late embryogenesis stages are known to repress transcription of genes related to the synthesis of new proteins [38]. Similarly, there is increased expression of histone core genes (*h2a, h2b, h4…*) at the early embryo developmental stages than in late maturation stages [38], [40].

Overall analysis of these results shows that most genes clustering in overrepresentated functional categories display expression levels that are modulated along the embryo developmental process. Thus, we hypothetised that our 20DAP transgenic and conventional embryos might have slight differences in their precise developmental stage, and these small differences in embryo development could explain these observed transcriptome changes, especially for these genes whose expression is known to rapidly change during embryo mid-development. The expression pattern of all 140 genes showing different expression in DKC6576 and Tietar 20 DAP embryos were *in silico* assessed using the publicly available tissue-specific expression atlas of maize [40]. Nine genes up-regulated and 10 genes down-regulated in the MON810 variety were expressed at different levels in embryos at 16, 18, 20, 22 and 24 DAP (Figure S5 A and B respectively). All 9 genes over-expressed in DKC6575 have stronger expression in the early developmental phases (16–20 DAP) than in more mature embryos. Conversely, all 10 genes expressed at lower levels in MON810 than in near-isogenic 20 DAP embryos display higher expression levels at late developmental stages (20–24 DAP) than in young embryos (Figure S5). This strongly suggests that seed development of MON810 variety DKC6575 is slightly delayed compared to Tietar variety. This is in agreement with different levels of ABA at 50 DAP and mature embryos, although they were not observed at early developmental stages. In addition, some minor physiological differences were observed between MON810 and conventional seeds. In particular, germination of mature DKC6575 seeds was quicker than that of Tietar (Figure S4). In addition, 20 DAP MON810 embryos consistently had lower dry weight, axis length and total area than near-isogenic embryos at the same stage (Figure 4). However, these differences seem to disappear at full maturity (10% grain moisture). Previous studies have demonstrated that maize embryos dry weight increases 6-10-fold at early embryogenesis stages (until 25 DAP) [44], [45]. These results together with the showed molecular data indicate a slower developmental timing of MON810 plants at reproductive stages. This delayed maturity phenotype is also observed in fields, where MON810 plants present a “stay-green” phenotype at later maturity stages with delayed senescence [46]

Transgenic maize event MON810 is worldwide commercialised in many different varieties obtained by conventional breeding, each providing a different genetic background that defines its specific characteristics. As a result, commercial varieties of the MON810 event may have different similarity levels to their near-isogenic counterparts [7], even if a set of sequences is differentially expressed in various variety pairs. We addressed the possible preservation of the transcriptomic differences identified in 20 DAP embryos of DKC6575 and Tietar by RT-qPCR quantification of the mRNA levels of up to 30 genes in a total of three MON810 and near-isogenic variety pairs, i.e. DKC6575 and Tietar; DKC6041-YG and DKC6040, and PR33P67 and PR33P66, the latter bred by a different company. Thirteen percent of these genes were specifically regulated in DKC6575 and Tietar, thus their transcriptional modulation could not be directly attributed to the transgene, but these unintended effects would most probably depend on the genetic background of this specific variety pair, as previously suggested [7]. Nevertheless, and although we cannot discard that they are similarly expressed in other pairs of transgenic and conventional comparable varieties, roughly 75% tested genes were differentially expressed in the three analysed variety pairs. They have different chromosomal locations, thus discarding their collective regulation due to poor segregation versus the transgene along the different breeding programs; and display different functions, making it unlikely that this phenomenon directly arises from the transgene insertion or the altered product of a gene located near the transgene.

### Characterisation of the transgenic insert and the *zm-upl* flanking the transgene

The MON810 transgene insertion resulted in truncation of an endogenous HECT type E3 ubiquitin ligase gene (*zm-upl*) [4], [5]. HECT type 3 ubiquitin ligases are involved in targeting specific protein substrates to proteasome srtructures for degradation [41], [42]. Our RT-qPCR results that the expression of the endogenous *zm-upl* gene is inhibited in a *cryIA(b)* homozygous line obtained in our greenhouses, but it is not reduced in commercial hemizygotic lines (Figure 4). Thus, truncation of *zm-upl* due to transgene insertion does not seem to be responsible for the observed differences in the transcriptome, although an effect on specific tissues cannot be discarded. Note that the genomic rearrangement associated with the MON810 transgene insertion has not been studied in detail, therefore we cannot rule out that it may have an effect on other sequences in the same locus.

### Conclusions

In conclusion, overall transcription is similar in 20 DAP embryos of the MON810 variety DKC6575 and the corresponding near-isogenic variety Tietar. Nevertheless, about 140 genes had altered transcription levels. Upon global analysis of their biological functions and known transcription patterns along embryogenesis and in response to the hormone ABA, their changed expression seems to be indicative of small differences in seed development in MON810 versus conventional comparators. MON810 appears to have slightly delayed maturation processes than conventional varieties, which includes embryo development and e.g. the delayed senescence phenotype observed in commercially grown MON810 plants (stay-green phenotype) [43]. These observed differences in transcription are most probably linked to the MON810 event but are not associated to undesirable changes in the global phenotype and plant behaviour, nor in the chemical and nutritional composition. We also found minor differences that are essentially attributed to the varietal genetic background, and are probably not directly related to the genetic modification. It should be noticed that the occurrence of unintended effects is not unique to the application of recombinant DNA techniques but has also been frequently observed in traditional breeding due to hybridisation, natural genetic recombination, natural chromosomal rearrangements or activity of transposable elements in plant genomes.

## Supporting Information

Figure S1 **Schematic diagram of the construction of 3′UTR-anchored poly-A(+) sscDNA libraries for 454 mRNA-seq analysis of DKC6575 and Tietar 20 DAP embryos.**
(TIF)

Figure S2 **MA-plots summarising the mRNA-seq (using DEseq, A; and EdgeR, B) and microarray hybridization (C) based comparison of DKC6575 and Tietar 20 DAP embryo transcriptomes.** The green line corresponds to the normalised distribution of counts.
(TIF)

Figure S3 **Levels of ABA in 20 DAP (A), 50 DAP (B) and mature (C) embryos of MON810 (blue) and near-isogenic (red) maize varieties.** ABA concentrations were measured using the AGdia ELISA kit and are expressed in ng ABA/g of fresh weight. Means and SD of three analytical replicates are shown. Asterisk shows statistically different ABA values (one-way ANOVA, Tukey's post-test α<0.05).
(TIF)

Figure S4 **Monitoring of** ***In vitro*** **germination of MON810 (var. DKC6575, A and C) and its near-isogenic counterpart (var. Tietar, B and D).** Radicle (A and B) and plumule (C and D) lengths were measured in 240 seed per variety along 9 consecutive days. Length values were grouped into categories (0, <10, 10–30 and >30 mm), see legend.
(TIF)

Figure S5 ***In silico*** **expression of 9 upregulated genes between DKC6575 and Tietar (A), and 10 down-regulated genes (B), at 16 to 20 DAP stages (blue bars), and 22 to 24 DAP stages (red bars).** Expression values are in log intensity of hibridization according to the maize expression atlas from [39]. Up-regulated genes in MON810 have higher expression in early maturation stages (A), while down-regulated ones have higher expression in late maturation stages (B).
(TIF)

Table S1 **Candidate differential expressed genes between MON810 and near-isogenic varieties identified by 454-mRNAseq and microarray.**
(DOCX)

Table S2 **Genes showing differential expression in DKC6575 and Tietar 20 DAP embryos, as identified using mRNA-seq coupled to the DEseq package (labeled in blue are genes up-regulated in MON810 embryos; labeled in red are those down-regulated in MON810 embryos).**
(XLSX)

Table S3 **Genes showing differential expression in DKC6575 and Tietar 20 DAP embryos, as identified using mRNA-seq coupled to the EdgeR package (labeled in blue are genes up-regulated in MON810 embryos; labeled in red are those down-regulated in MON810 embryos).**
(XLSX)

Table S4 **Genes showing differential expression in DKC6575 and Tietar 20 DAP embryos, as assessed by microarray hybridization (labeled in blue are genes up-regulated in MON810 embryos; labeled in red are those down-regulated in MON810 embryos).**
(XLSX)

Table S5 **qPCR validation of 30 selected genes, 15 upregulated and 15 downregulated. Log2 FC (log2 fold change).**
(DOCX)

Table S6 **mRNA-seq reads matching** ***cry1A(b)*** **coding sequence in MON810 maize, variety DKC6575.**
(DOCX)

Table S7 **Oligonucleotides used in this study.** Bold-underlined sequences are meant to directionally ligate 454 pyrosequencing adaptors.
(DOCX)

Table S8 **Morphological measurements (mean ± S.E.) of dry weight, axis length and embryo area for the MON810 – near isogenic variety pairs DKC6575-Tietar, PR33P67-PR33P66 and DKC6041YG-DKC6040.** Statistical analysis of means (t-test p-value, significance level of 0.05) for each parameter is show on the lower part of the table.
(DOCX)
