# Supplementary figures and images for: The Use of Massive Sequencing to Detect Differences between Immature Embryos of MON810 and a Comparable Non-GM Maize Variety
Source: PLoS One. 2014 Jun 26;9(6):e100895. doi: 10.1371/journal.pone.0100895 (PMC4072715; doi:10.1371/journal.pone.0100895)

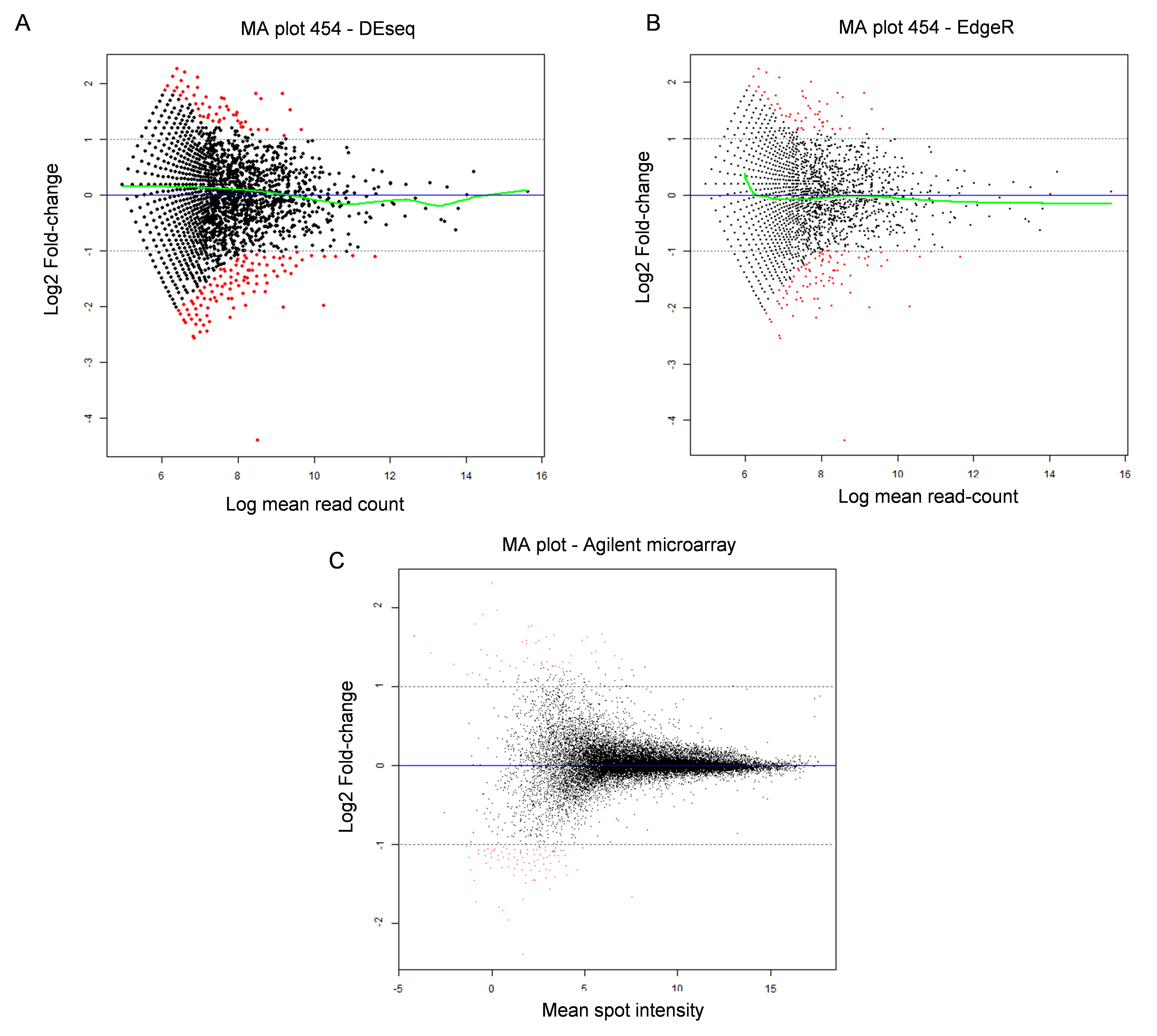

Supplement: Figure S2 — MA-plots summarising the mRNA-seq (using DEseq, A; and EdgeR, B) and microarray hybridization (C) based comparison of DKC6575 and Tietar 20 DAP embryo transcriptomes. The green line corresponds to the normalised distribution of counts. (TIF) [file pone.0100895.s002.tif]

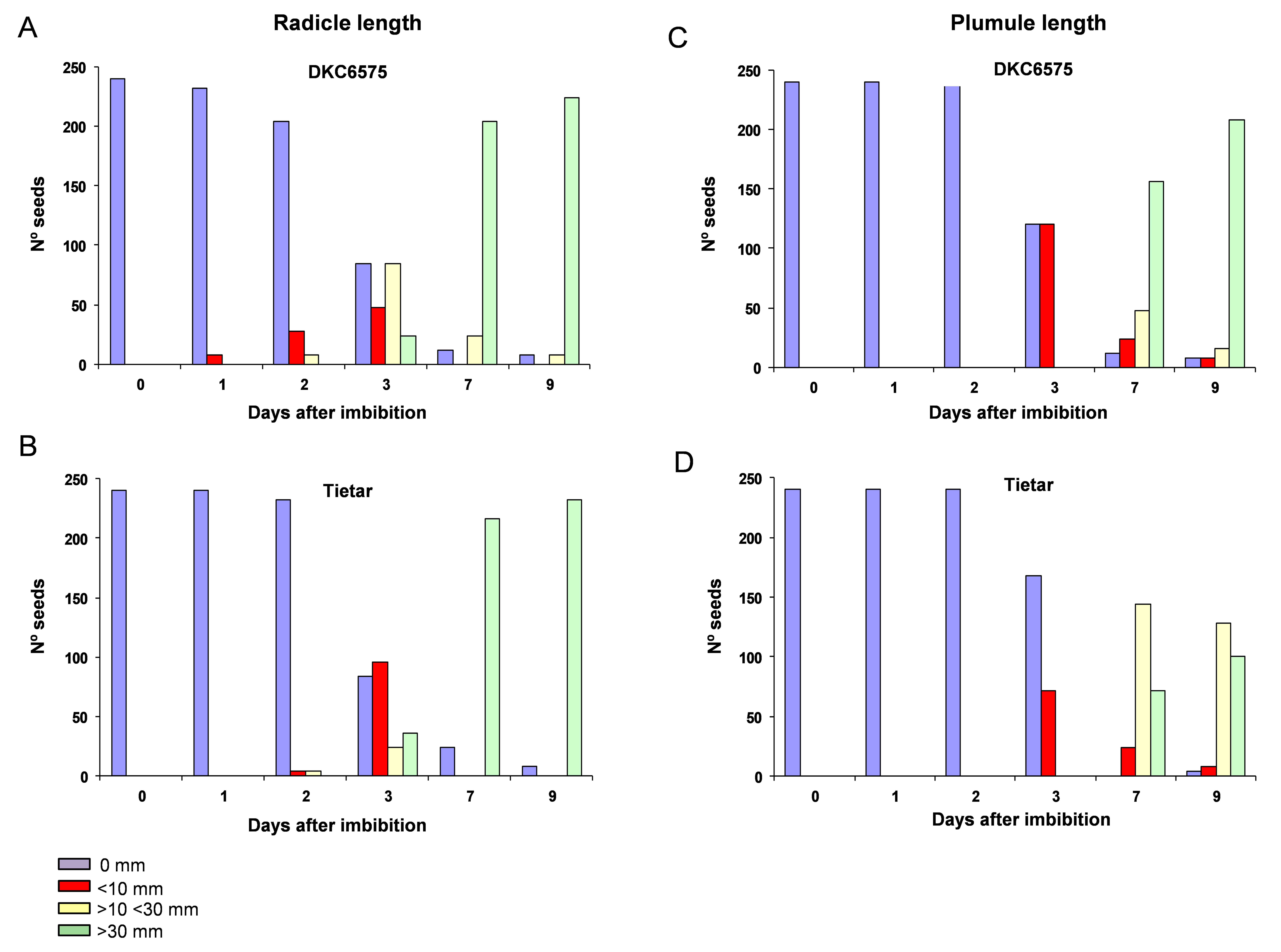

Supplement: Figure S4 — Monitoring of In vitro germination of MON810 (var. DKC6575, A and C) and its near-isogenic counterpart (var. Tietar, B and D). Radicle (A and B) and plumule (C and D) lengths were measured in 240 seed per variety along 9 consecutive days. Length values were grouped into categories (0, <10, 10–30 and >30 mm), see legend. (TIF) [file pone.0100895.s004.tif]
