# Supplementary material for: The Use of Massive Sequencing to Detect Differences between Immature Embryos of MON810 and a Comparable Non-GM Maize Variety
Source: PLoS One. 2014 Jun 26;9(6):e100895. doi: 10.1371/journal.pone.0100895 (PMC4072715; doi:10.1371/journal.pone.0100895)
Supplement: Table S1 — Candidate differential expressed genes between MON810 and near-isogenic varieties identified by 454-mRNAseq and microarray. (DOCX) [file pone.0100895.s006.docx]

**Table S1**. Candidate differential expressed genes between MON810 and near-isogenic varieties identified by 454-mRNAseq and microarray.

|  | **Log2-Fold-change** | | |  |  |
| --- | --- | --- | --- | --- | --- |
| **Maize Gene ID** | **DEseq** | **EdgeR** | **microarray** | **Description** | **GO: Biological process** |
| GRMZM2G466833 | 2.2665 | 2.2665 | NA | Malate dehydrogenase (Z.mays) | CHO metabolism |
| GRMZM5G806622 | 2.2665 | 2.2665 | NA | DUF putative uncharacterized protein (O. sativa) | Unknown |
| GRMZM2G031501 | 2.1961 | 2.1961 | 2.0753 | Uncharacterized protein (Z.mays) | Unknown |
| GRMZM2G477205 | 2.1221 | 2.1221 | 1.7266 | E3-Ubiquitin ligase related (A.thaliana) | Protein modification |
| GRMZM2G073934 | 2.1030 | 2.1030 | NA | mitocondrial associated protein (O.sativa) | Protein modification |
| GRMZM2G048455 | 2.0172 | 2.0172 | NA | MPK7-putative kinase (Z.mays) | Protein modification |
| GRMZM2G015610 | 1.8591 | 1.8482 | 1.5521 | Uncharacterized protein (Z.mays) | Unknown |
| GRMZM2G022041 | 1.8591 | 1.8591 | 1.5521 | Ribonucleoprotein-associated protein (Z.mays) | Protein biosynthesis |
| GRMZM2G065908 | 1.8482 | 1.8591 | NA | Uncharacterized protein (Z.mays) | Unknown |
| GRMZM2G153227 | 1.8482 | 1.8482 | 1.7057 | H/ACA ribonucleoprotein complex subunit 3-like (Z.mays) | Protein biosynthesis |
| GRMZM2G325575 | 1.8482 | 1.8482 | 1.7438 | Ferritin-1, chloroplastic (Z.mays) | Oxidorreduction |
| GRMZM2G415359 | 1.8482 | 1.8482 | NA | Malate dehydrogenase, cytoplasmic (Z.mays) | CHO metabolism |
| GRMZM2G015605 | 1.8163 | 1.8163 | 1.1811 | dehydration protein putative (Z.mays) | Stress response |
| GRMZM5G867256 | 1.8135 | 1.8135 | NA | Uncharacterized protein (Z.mays) | Unknown |
| GRMZM2G040095 | 1.7811 | 1.7811 | 1.7409 | Lipoxygenase (Z.mays) | Lipid metabolism |
| GRMZM2G146697 | 1.7811 | 1.7811 | 1.5490 | Uncharacterized protein (Z.mays) | Unknown |
| GRMZM2G164352 | 1.7811 | 1.7811 | 1.3096 | Protein phosphatase 2A regulatory subunit A (Z.mays) | Protein modification |
| GRMZM2G172369 | 1.7811 | 1.7811 | NA | Mannose binding-protein (Z.mays) | CHO metabolism |
| GRMZM2G436835 | 1.7811 | 1.7811 | 1.0078 | Uncharacterized protein (Z.mays) | Unknown |
| GRMZM2G093325 | 1.7338 | 1.7338 | NA | Early response to dehydration-15 (A.thaliana) | Stress response |
| GRMZM2G008327 | 1.7197 | 1.7197 | 1.1240 | Cell division control protein 2 homolog (Z.mays) | Cell cycle |
| GRMZM2G013811 | 1.7197 | 1.7197 | NA | 4-alpha-glucanotransferase putative (Z.mays) | CHO metabolism |
| GRMZM2G025783 | 1.7197 | 1.7197 | 1.7505 | Protein kinase Kelch repeat:Kelch (Z.mays) | Protein modification |
| GRMZM2G059138 | 1.6618 | 1.6618 | NA | SAUR33-auxin-responsive family member (Z.mays) | Transcription |
| GRMZM5G802816 | 1.5808 | 1.5808 | 1.0664 | Uncharacterized protein (Z.mays) | Unknown |
| GRMZM2G316362 | 1.5229 | 1.5229 | 1.2735 | Acyl-desaturase (Z.mays) | Lipid metabolism |
| GRMZM2G425482 | 1.4799 | 1.4799 | NA | Uncharacterized protein (Z.mays) | Unknown |
| GRMZM2G122481 | 1.4440 | 1.4440 | 1.3024 | Cytochrome C oxidase (Z.mays) | Oxidorreduction |
| GRMZM2G106511 | 1.4371 | 1.4371 | NA | Uncharacterized protein (Z.mays) | Unknown |
| GRMZM2G152891 | 1.4274 | 1.4274 | NA | Calmodulin (Z.mays) | Signal transduction |
| GRMZM2G137558 | 1.4012 | 1.4012 | NA | Uncharacterized protein (Z.mays) | Unknown |
| GRMZM2G082484 | 1.3745 | 1.3745 | 1.3515 | Uncharacterized protein (Z.mays) | Unknown |
| GRMZM2G107116 | 1.3481 | 1.3481 | 1.7395 | Proteasome activator subunit 4 like (Z.mays) | Protein modification |
| GRMZM2G028955 | 1.3336 | 1.3336 | NA | Histone H2A6 (Z.mays) | Chromatin organization |
| GRMZM2G118637 | 1.3154 | 1.3154 | NA | Putative ubiquitin family protein isoform 1 (Z.mays) | Protein modification |
| GRMZM2G421279 | 1.2992 | 1.2992 | NA | Histone H4C14 (Z.mays) | Chromatin organization |
| GRMZM2G072855 | 1.2741 | 1.2741 | NA | Histone H4C7 (Z.mays) | Chromatin organization |
| GRMZM2G003306 | 1.2217 | 1.2217 | NA | Histone H2A12 (Z.mays) | Chromatin organization |
| GRMZM2G037177 | 1.2208 | 1.2208 | NA | Gamma carbonic anhydrase 2 (O.sativa) | Stress response |
| GRMZM2G160506 | 1.1804 | 1.1804 | NA | Uncharacterized protein (Z.mays) | Unknown |
| GRMZM2G305362 | 1.1775 | 1.1775 | 1.0389 | Chromatin-binding protein (Z.mays) | Chromatin organization |
| GRMZM2G181607 | 1.1747 | 1.1747 | 2.8618 | Uncharacterized protein (Z.mays) | Unknown |
| GRMZM2G359038 | 1.1707 | 1.1707 | 1.3065 | Uncharacterized protein (Z.mays) | Unknown |
| GRMZM2G181153 | 1.1417 | 1.1417 | NA | Histone H4C13 (Z.mays) | Chromatin organization |
| GRMZM2G028929 | 1.1395 | 1.1395 | 1.0654 | Epsilon-COP (Z.mays) | Transport |
| GRMZM2G061135 | 1.0741 | 1.0741 | 1.0004 | S-adenosylmethionine synthase (Z.mays) | Protein modification |
| GRMZM2G151826 | 1.0706 | 1.0706 | 1.5074 | Histone H2A2 (Z.mays) | Chromatin organization |
| GRMZM2G096435 | 1.0094 | 1.0094 | 1.0306 | Oleosin Bn-V (Z.mays) | Storage |
| GRMZM2G097229 | -1.0044 | -1.0044 | NA | Expansin B4 (O.sativa) | CHO metabolism - Cell wall |
| GRMZM2G102230 | -1.0150 | -1.0150 | NA | 60S ribosomal protein L23 (Z.mays) | Ribosomal consituent |
| GRMZM2G088212 | -1.0291 | -1.0291 | -1.0980 | Catalase isozyme 1 (Z.mays) | Oxidorreduction |
| GRMZM2G415007 | -1.0669 | -1.0669 | NA | Luminal-binding protein 3 (Z.mays) | ATP biosynthesis |
| GRMZM2G104632 | -1.0956 | -1.1050 | NA | GADPH, cytosolic (Z.mays) | CHO metabolism |
| GRMZM2G327564 | -1.1050 | -1.0956 | -1.1659 | 60S ribosomal protein L26-1 (Z.mays) | Ribosomal consituent |
| GRMZM2G118003 | -1.1056 | -1.1056 | NA | Cellulase (O.sativa) | CHO metabolism - Cell wall |
| GRMZM2G026991 | -1.1197 | -1.1197 | -1.1957 | Uncharacterized protein (Z.mays) | Unknown |
| GRMZM2G102891 | -1.1258 | -1.1258 | -1.4131 | 60S acidic ribosomal protein (Z.mays) | Ribosomal consituent |
| GRMZM2G095593 | -1.1369 | -1.1369 | -1.1895 | 26S protease regulatory subunit 4 homolog (O.sativa) | Protein modification |
| GRMZM5G813584 | -1.1497 | -1.1497 | NA | 60S ribosomal protein L11 (Z.mays) | Ribosomal consituent |
| GRMZM2G117642 | -1.1591 | -1.1591 | -1.2255 | Uncharacterized protein (Z.mays) | Unknown |
| GRMZM2G174757 | -1.1872 | -1.1872 | -1.3206 | Eukaryotic translation initiation factor 3 subunit B (Z.mays) | Protein biosynthesis |
| GRMZM2G324314 | -1.1909 | -1.1909 | NA | 60S ribosomal protein L13 (Z.mays) | Ribosomal consituent |
| GRMZM2G135727 | -1.2189 | -1.2189 | -1.3509 | 60S ribosomal protein L3 (O.sativa) | Ribosomal consituent |
| GRMZM2G076544 | -1.2400 | -1.2400 | -1.8485 | Peptidyl-prolyl cis-trans isomerase (Z.mays) | Protein biosynthesis |
| GRMZM2G158568 | -1.2504 | -1.2513 | -1.5481 | 60S ribosomal protein L31 (Z.mays) | Ribosomal consituent |
| GRMZM2G179981 | -1.2513 | -1.2513 | -1.2448 | Putative cinnamyl alcohol dehydrogenase (O.sativa) | Secondary metabolism |
| GRMZM2G326545 | -1.2513 | -1.2504 | -1.0335 | Uncharacterized protein (Z.mays) | Unknown |
| GRMZM2G101287 | -1.3274 | -1.3274 | NA | Pathogen induced protein 2-4 (Z.mays) | Stress response |
| GRMZM2G416388 | -1.3311 | -1.3311 | -1.2754 | CBS domain protein (Z.mays) | Oxidorreduction |
| GRMZM2G167637 | -1.3514 | -1.3514 | -1.0637 | Pectinesterase (Z.mays) | CHO metabolism - Cell wall |
| GRMZM2G123371 | -1.3528 | -1.3528 | -1.0765 | Uncharacterized protein (Z.mays) | Unknown |
| GRMZM2G099754 | -1.3541 | -1.3541 | NA | Serine/threonine-protein kinase (Z.mays) | Protein modification |
| GRMZM5G870606 | -1.3603 | -1.3603 | NA | Putative MYB DNA-binding protein (Z.mays) | Transcription |
| GRMZM2G105364 | -1.4116 | -1.4116 | NA | Uncharacterized protein (Z.mays) | Unknown |
| GRMZM2G137139 | -1.4443 | -1.4443 | NA | Uncharacterized protein (Z.mays) | Unknown |
| GRMZM2G100146 | -1.4516 | -1.4516 | NA | Histone deacetylase HDT2 (Z.mays) | Protein modification |
| GRMZM2G002178 | -1.4668 | -1.4668 | -1.5967 | Cytochrome P450 CYP74A19 (Z.mays) | Oxidorreduction |
| GRMZM2G310002 | -1.4668 | -1.4668 | NA | Uncharacterized protein (Z.mays) | Unknown |
| GRMZM2G115674 | -1.4701 | -1.4701 | NA | Uncharacterized protein (Z.mays) | Unknown |
| GRMZM2G174896 | -1.4857 | -1.4857 | NA | CIPK-like protein 1 (Z.mays) | Transport |
| GRMZM2G447795 | -1.5332 | -1.5332 | -1.4344 | Xylanase inhibitor protein 1 (Z.mays) | CHO metabolism - Cell wall |
| GRMZM2G140201 | -1.5332 | -1.5343 | NA | Endo-Beta-Mannanase (O.sativa) | CHO metabolism |
| GRMZM2G302131 | -1.5343 | -1.5332 | -1.2454 | Uncharacterized protein (Z.mays) | Unknown |
| GRMZM2G075683 | -1.5539 | -1.5539 | -1.0170 | Casein-kinase 1 CK1 like (O.sativa) | Protein modification |
| GRMZM2G052088 | -1.5744 | -1.5744 | -1.0447 | Vacuolar ATP synthase subunit d, putative (O.sativa) | Transport |
| GRMZM2G069542 | -1.5841 | -1.5841 | NA | Phosphoenolpyruvate carboxylase (Z.mays) | CHO metabolism |
| GRMZM2G007195 | -1.5881 | -1.5881 | -1.1052 | UDP-glucuronic acid decarboxylase putative (O.sativa) | CHO metabolism |
| GRMZM2G100120 | -1.5974 | -1.5974 | -1.5544 | eIF3 subunit K (Z.mays) | Protein synthesis |
| GRMZM2G028286 | -1.6184 | -1.6184 | -1.2257 | Xyloglucan glycosyltransferase 10 (O.sativa) | CHO metabolism - Cell wall |
| GRMZM2G149717 | -1.6220 | -1.6220 | NA | Uncharacterized protein (Z.mays) | Unknown |
| GRMZM2G702490 | -1.6270 | -1.6270 | -1.2210 | Propionyl-CoA carboxylase beta chain (Z.mays) | Aminoacid metabolism |
| GRMZM2G027282 | -1.6340 | -1.6340 | NA | 26S protease regulatory subunit 6A, putative (O.sativa) | Protein modification |
| GRMZM2G083173 | -1.6404 | -1.6404 | -1.6644 | Hexose transporter (Z.mays) | CHO metabolism |
| GRMZM2G051256 | -1.6519 | -1.6519 | NA | MYB-IF35 transcription factor (Z.mays) | Transcription |
| GRMZM2G011575 | -1.7108 | -1.7108 | NA | Uncharacterized protein (Z.mays) | Unknown |
| GRMZM2G164759 | -1.7108 | -1.7108 | -1.5269 | Uncharacterized protein (Z.mays) | Unknown |
| GRMZM2G385989 | -1.7214 | -1.7214 | -1.2205 | Vesicle-associated membrane protein (Z.mays) | Transport |
| GRMZM5G863602 | -1.7214 | -1.7214 | -2.3065 | Senescence-associated protein DH (Z.mays) | Cell cycle |
| GRMZM2G176340 | -1.7368 | -1.7368 | NA | F-box and tubby domain containing protein (Z.mays) | Cell cycle |
| GRMZM2G101001 | -1.7403 | -1.7514 | -1.0327 | Uncharacterized protein (Z.mays) | Unknown |
| GRMZM2G134251 | -1.7487 | -1.7514 | -1.0714 | Beta-hexosaminidase (Z.mays) | CHO metabolism - Cell wall |
| GRMZM2G046191 | -1.7514 | -1.7403 | NA | Putative tryptophan synthase alpha (Z.mays) | Aminoacid biosynthesis |
| GRMZM2G141665 | -1.7514 | -1.7487 | -1.2084 | Syringomycin biosynthesis enzyme (Z.mays) | Oxidorreduction |
| GRMZM2G375002 | -1.7632 | -1.7632 | NA | Rac-like GTP-binding protein 5 (Z.mays) | Protein modification |
| GRMZM2G027862 | -1.7811 | -1.7811 | -1.0111 | Cellulose synthase-interactive protein 1 (A.thaliana) | CHO metabolism - Cell wall |
| GRMZM2G076062 | -1.8363 | -1.8363 | NA | Uncharacterized protein (Z.mays) | Unknown |
| GRMZM2G071333 | -1.8483 | -1.8483 | -1.0995 | leucyl-tRNA synthetase, putative (O.sativa) | Protein biosynthesis |
| GRMZM5G898915 | -1.8512 | -1.8628 | -1.3694 | Aldose 1-epimerase, putative (O.sativa) | CHO metabolism |
| GRMZM2G310431 | -1.8545 | -1.8512 | -7.8624 | Heat shock 70 kDa protein (Z.mays) | Stress response |
| GRMZM2G407044 | -1.8545 | -1.8545 | -1.4728 | Acetolactate synthase (Z.mays) | Aminoacid metabolism |
| GRMZM2G134797 | -1.8628 | -1.8545 | -1.1624 | Nucleoside diphosphate kinase (Z.mays) | Protein modification |
| GRMZM2G127117 | -1.8742 | -1.8743 | -1.5134 | Glucan endo-1,3-beta-glucosidase 7 (Z.mays) | CHO metabolism - Cell wall |
| GRMZM2G003028 | -1.8743 | -1.8743 | -1.1322 | Thioredoxin, putative (O.sativa) | Oxidorreduction |
| GRMZM2G057448 | -1.8743 | -1.8743 | -1.2316 | Histone-arginine methyltransferase CARM1 (Z.mays) | Protein modification |
| GRMZM2G163297 | -1.8743 | -1.8743 | NA | ELAV-like protein 4 (Z.mays) | Transcription |
| GRMZM2G165817 | -1.8743 | -1.8743 | -1.7578 | 26S proteasome regulatory ATPase subunit 4 (Z.mays) | Protein modification |
| GRMZM2G396248 | -1.8743 | -1.9034 | NA | Putative cytochrome P450 superfamily protein (Z.mays) | Oxidorreduction |
| GRMZM2G342327 | -1.8990 | -1.8990 | NA | 2-phosphoglycerate kinase, putative (O.sativa) | CHO metabolism |
| GRMZM2G130987 | -1.9034 | -1.9414 | -1.9912 | Sec61 subunit alpha transport protein (O.sativa) | Transport |
| GRMZM2G366935 | -1.9414 | -1.9507 | -1.8286 | Serine/threonine-protein kinase AFC3, putative (Z.mays) | Protein modification |
| GRMZM2G010095 | -1.9507 | -1.9738 | -1.0946 | Uncharacterized protein (Z.mays) | Unknown |
| GRMZM2G011253 | -1.9738 | -1.9738 | -1.1634 | 60S ribosomal protein L31 (Z.mays) | Ribosomal consituent |
| GRMZM2G079538 | -1.9738 | -1.9738 | NA | 2-oxoglutarate dehydrogenase complex E2 (Z.mays) | CHO metabolism |
| GRMZM2G137348 | -1.9738 | -2.0055 | -1.2214 | Uncharacterized protein (Z.mays) | Unknown |
| GRMZM2G330635 | -1.9824 | -2.0055 | -1.4677 | Glutathione S-transferase GSTU6 (Z.mays) | Oxidorreduction |
| GRMZM2G018375 | -2.0055 | -1.8742 | -1.2194 | Thiamine thiazole synthase 1, chloroplastic (Z.mays) | Stress response |
| GRMZM2G068586 | -2.0055 | -2.0055 | -1.3229 | TUB transcription factor (Z.mays) | Transcription |
| GRMZM2G074957 | -2.0055 | -1.9824 | NA | Kinesin light chain-like protein (Z.mays) | Protein modification |
| GRMZM2G130052 | -2.0055 | -2.0055 | -1.5408 | Uncharacterized protein (Z.mays) | Unknown |
| GRMZM2G412470 | -2.0189 | -2.0189 | -1.3886 | MA3 domain containing protein (Z.mays) | RNA metabolism |
| GRMZM2G044004 | -2.0409 | -2.0409 | -1.0237 | Nucleic acid binding protein (Z.mays) | Transcription |
| GRMZM2G139374 | -2.0669 | -2.0669 | NA | Uncharacterized protein (Z.mays) | Unknown |
| GRMZM2G111143 | -2.1258 | -2.1258 | -1.3414 | Glucan endo-1,3-beta-glucosidase (O.sativa) | CHO metabolism - Cell wall |
| GRMZM2G160032 | -2.1614 | -2.1614 | -1.5479 | Transcription factor X1, putative (Z.mays) | Transcription |
| GRMZM2G400718 | -2.1962 | -2.1962 | NA | Uncharacterized protein (Z.mays) | Unknown |
| GRMZM2G095657 | -2.2368 | -2.2368 | -1.8122 | Uncharacterized protein (Z.mays) | Unknown |
| GRMZM2G435338 | -2.2893 | -2.2893 | NA | 60S ribosomal protein L11-1 (Z.mays) | Ribosomal consituent |
| GRMZM2G000264 | -2.5263 | -2.5263 | -1.0626 | Plasma membrane ATPase 11, putative (O.sativa) | ATP biosynthesis |
| GRMZM2G086801 | -2.5694 | -2.5694 | -1.0394 | COP9 signalosome complex subunit 5b (Z.mays) | Transport |
| GRMZM2G056252 | -4.3888 | -4.3888 | NA | Fatty acid desaturase, putative (O.sativa) | Lipid metabolism |

*N.A. = not analyzed
