# Supplementary material for: The Use of Massive Sequencing to Detect Differences between Immature Embryos of MON810 and a Comparable Non-GM Maize Variety
Source: PLoS One. 2014 Jun 26;9(6):e100895. doi: 10.1371/journal.pone.0100895 (PMC4072715; doi:10.1371/journal.pone.0100895)
Supplement: Table S5 — qPCR validation of 30 selected genes, 15 upregulated and 15 downregulated. Log2 FC (log2 fold change). (DOCX) [file pone.0100895.s010.docx]

**Table S5**. qPCR validation of 30 selected genes, 15 upregulated and 15 downregulated. Log2 FC (log2 fold change).

| **Maize Gene ID** | **log2 FC** | ***p-value*** | **Description** | **GM vs non-GM** |
| --- | --- | --- | --- | --- |
| GRMZM2G466833 | 2.3722 | 3.1613E-13 | Malate dehydrogenase | Upregulated |
| GRMZM2G027862 | 2.3084 | 2.4315E-06 | Cellulose synthase 1 | Upregulated |
| GRMZM2G118637 | 1.4256 | 3.2592E-14 | Putative ubiquitin family protein isoform 1 | Upregulated |
| GRMZM2G107116 | 1.3481 | 1.9037E-04 | Proteasome activator subunit 4 putative | Upregulated |
| GRMZM2G477205 | 2.1221 | 1.0667E-03 | E3-Ubiquitin ligase related | Upregulated |
| GRMZM2G013811 | 1.8321 | 5.5414E-03 | 4-alpha-glucanotransferase putative | Upregulated |
| GRMZM2G172369 | 1.9822 | 9.0188E-03 | Mannose binding-protein | Upregulated |
| GRMZM2G028955 | 1.4820 | 3.9389E-03 | Histone H2A6 | Upregulated |
| GRMZM2G421279 | 1.4178 | 1.5670E-03 | Histone H4C14 | Upregulated |
| GRMZM2G072855 | 1.5128 | 4.4374E-02 | Histone H4C7 | Upregulated |
| GRMZM2G181153 | 1.3904 | 8.1205E-03 | Histone H4C13 | Upregulated |
| GRMZM2G151826 | 1.3105 | 1.0667E-03 | Histone H2A2 | Upregulated |
| GRMZM2G093325 | 1.8723 | 9.7791E-05 | Early response to dehydration-15 | Upregulated |
| GRMZM2G015605 | 1.1144 | 1.4988E-06 | Dehydration protein putative | Upregulated |
| GRMZM2G040095 | 1.1216 | 1.1509E-09 | Lipoxygenase | Upregulated |
| GRMZM2G097229 | -1.1430 | 1.5513E-18 | Expansin B4 | Downregulated |
| GRMZM2G102230 | -1.2241 | 1.0862E-17 | 60S ribosomal protein L23 | Downregulated |
| GRMZM2G327564 | -1.6322 | 9.6390E-17 | 60S ribosomal protein L26-1 | Downregulated |
| GRMZM2G118003 | -1.2846 | 3.2592E-14 | Cellulase | Downregulated |
| GRMZM2G167637 | -1.5287 | 2.3802E-13 | Pectinesterase | Downregulated |
| GRMZM2G140201 | -1.6996 | 4.7883E-12 | Endo-Beta-Mannanase | Downregulated |
| GRMZM2G071333 | -2.0000 | 6.5740E-09 | leucyl-tRNA synthetase | Downregulated |
| GRMZM2G046191 | -2.1423 | 3.1344E-07 | Putative tryptophan synthase alpha | Downregulated |
| GRMZM2G083173 | -2.5356 | 1.5384E-05 | Hexose transporter | Downregulated |
| GRMZM2G018375 | -2.4030 | 3.2813E-04 | Thiamine thiazole synthase 1 | Downregulated |
| GRMZM2G407044 | -1.9695 | 6.4033E-03 | Acetolactate synthase | Downregulated |
| GRMZM2G179981 | -1.7884 | 2.1533E-04 | Putative cinnamyl alcohol dehydrogenase | Downregulated |
| GRMZM2G028286 | -1.9265 | 3.3503E-02 | Xyloglucan glycosyltransferase 10 | Downregulated |
| GRMZM2G447795 | -1.8206 | 1.9037E-04 | Xylanase inhibitor protein 1 | Downregulated |
| GRMZM2G134251 | -2.7549 | 8.8813E-04 | Beta-hexosaminidase | Downregulated |
