# Supplementary material for: The Use of Massive Sequencing to Detect Differences between Immature Embryos of MON810 and a Comparable Non-GM Maize Variety
Source: PLoS One. 2014 Jun 26;9(6):e100895. doi: 10.1371/journal.pone.0100895 (PMC4072715; doi:10.1371/journal.pone.0100895)
Supplement: Table S6 — mRNA-seq reads matching cry1A(b) coding sequence in MON810 maize, variety DKC6575. (DOCX) [file pone.0100895.s011.docx]

**Table 6**. mRNA-seq reads matching *cry1A(b)* coding sequence in MON810 maize, variety DKC6575.

| **454-read ID** | **Sequence** |
| --- | --- |
| >GI54YUC01ET2TR | CGACGAGAGGAATCTCCTCCAGGACCCCCAATTTTCCGCGGCATCAACAGGCAGCTCGACCGCGGCTGGCGCGGCAGCACCGACATCACGATCCAGGGGCGGCGACGATGTGTTCAAGGAGAACTACGTGACTCTCCTGGGCACTTTCGACGAGTGCTACCCTACCTACTTGTACCAGAAGATCGATGAGTCCAAGCTCAAGGCTTACACTCGCTACCAGCTCCGCGGCTACATCGAAGACAGCCAAGACCTCGAGATTTACCTGATCCGCTACAACGCCAAGCACGAGACCCGTCAACGTGCCCGGTCTTGGTT |
| >GI54YUC01CH3OJ | CCGGCTTCAGCAACTCGTCCGTGAGCATCATCAGAGCTCCTATGTTCTCCTGGATTCATCGCAGCGCGGAGTTCAACAATATCATTCCGTCCTCCCAAATCACCCAAATCCCCTCACCAAGTCCACCAACCTGGGCAGCGGCACCTCCGTGGTGAAGGGCCCAGGCTTCACGGGCGGCGACATCCTGCGCAGGACCTCCC |
| >GI54YUC01BRWFH | CCGGGCCAGATCAGCACCCTCCGCGTCAACATCACCGCTCCCCTGTCCCAGAGGTTACCGCGTTCAGGATTCGCTACGCTAGCACCACCAACCTGCAATTCCACACCTCCATCGACGGCAGGCCGATCAATCAGGGTAACTTCTCCGCCACCATGTCCAGCGGCAGCAACCTCCAATCCG |
| >GI54YUC01EENY0 | TCAATGAGCTCTTCACGTCCAGCAATCAGATCGGCCTGAAGACCGACGTCACTGACTACCACATCGACCAAGTCTCCAACCTCGTGGAGTGCCTCTCCGATGAGTTCTGCCTCGACGAGAAGAAGGAGCTGTCCGAGAAGGTGAAGCATGCCAAGCGTCTCAGCGA |
| >GI54YUC01BQDCW | TCAATGAGCTCTTCACGTCCAGCAATCAGATCGGCCTGAAGACCGACGTCACTGACTACCACATCGACCAAGTCTCCAACCTCGTGGAGTGCCTCTCCGATGAGTTCTGCCTCGACGAGAAGAAGGAGCTGTCCGAGAAGGTGAAGCATGCCAAGCGTCTCAGCG |
| >GI54YUC01C1EOF | TTCAGCGGCCCCCGAGTTTTACCCTTTTCCTCTCTACGGCACGATGGGGCAACGCCGCTCCACAACAACGCATCGTCGCTCAGCTGGGGCCAGGGGCGTCTACCGCACCCCTGAGCTCCACCCTGTACCGCAGGCCCTTCAACATCGGTATCAACAACCAGCAGCTGTCCGTCCTGGAATGGCACTGAGTT |
| >GI54YUC01BDG6S | GTGCTACCCTACCTACTTGTACCAGAAGATCGATGAGTCCAAGCTCAAGGCTTACACTCGCTACCAGCTCCGCGGCTACATCGAAGACAGCCAAGACCTCGAGATTTACCTGATCCGCTACAACGCCAAGCACGAGACCGTCAACGTGCCCGG |
| >GI54YUC01CG0Y6 | CCGGTACTGGTTCCCTCTGGCCGCTGAGCGCCCCCAGCCCGATCGGCAAGTGTGCCCACCACAGCCACCACTTCTCCTTGGACATCGATGTGGGCTGCACCGACCTGAACGAGGACTTTCGGTAG |
| >GI54YUC01DJIYX | CCGGTACTGGTTCCCTCTGGCCGCTGAGCGCCCCCAGCCCGATCGGCAAGTGTGCCCACCACAGTCACCACTTCTCCTTGGACATCGATGTGGGCTGCACCGACCTGAACGAGGACTTTCGGTAG |
| >GI54YUC01DIPSK | CGGTACTGGTTCCCTCTGGCCGCTGAGCGCCCCCAGCCCGATCGGCAAGTGTGCCCACCACAGCCACCACTTCTCCTTGGACATCGATGTGGGCTGCACCGACCTGAACGAGGACTTTCGGTAG |
| >GI54YUC01AS3PH | TCAATGAGCTCTTCACGTCCAGCAATCACGATCGGCCTCGAAGACCGACGTCACTGTACTTACCACAATCGACCAAGTCTCAACCTCGTGGAGTTGCCTCTCCGATTGAAGTTCTGACCTCGACGAGAAGAAGGACGCTGTCCGAGAAGGTGAACGACATGCCAAGCGTCTCAGCG |
| >GI54YUC01EFI0C | AGCTTCAGGGGCAGCGCCCAGGGCATCGAGGGCTCCATCAGGAGCCCACACCTGATGGACATCCTCAACAGCATCACTATCTACACCGATGCCCACCGCGGCGAGTACTACTGGTCCG |
| >GI54YUC01BM2ZM | CGGTACTGGTTCCCCTCTGGCCGCTGAGCGCCCCCCAGCCCGATCGGCAAGTGTGCCCACCACAGCCACCACTTCTCCTTGGACATCGATGTGGGCTGCACCGACCTGAACGAGGACTTTCGGTAG |
| >GI54YUC01BI23V | AGCTTCAGGGGCAGCGCCCAGGGCATCGAGGGCTCCATCAGGAGCCACACCTGATGGACATCCTCAACAGCATCACTATCTACACCGATG-CCACCGCGGCGAGTACTACTGGTCCG |
| >GI54YUC01B9TYI | AGCTCCTATGTTCTCCTGGGATTCATCGCAGCGCGGAGTTCAACAATATCATTCCGTCCTCCCAAAATCACCCCAAAATCCCCCCTCACCAAGTCCACCAACCTGGGC |
| >GI54YUC01AHMOF | GGCAAGTGTGCCCACCACAGCCACCACTTCTCCTTGGACATCGATGTGGGCTGCACCGACCTGAACGAGGACTTTCGGTAG |
| >GI54YUC01CA6BE | GGCAAGTGTGCCCACCACAGCCACCACTTCTCCTTGGACATCGATGTGGGCTGCACCGACCTGAACGAGGACTTTCGGTAG |
| >GI54YUC01A524Z | TCAACTTCTCCAACGGCTCCAGCGTTTTCACCCTGAGCGCCCACGTGTTCAATTCCGGCA |
| >GI54YUC01D82EZ | TCAACTTCTCCAACGGCTCCAGCGTTTTCACCCTGAGCGCCCACGTGTTCAATTCCGGC |
| >GI54YUC01AS6LA | TCAACTTCTCCAACGGCTCCAGCGTTTTCACCCTGAGCGCCCACGTGTTCAATTCCGG |
| >GI54YUC01EICXB | TCAACTTCTCCAACGGCTCCAGCGTTTTCACCCTGAGCGCCCACGTGTTCAATTCCG |
| >GI54YUC01D7EV0 | TCAACTTCTCCAACGGCTCCAGCGTTTTCACCCTGAGCGCCCACGTGTTCAATTCCG |
| > GI54YUC01BTQLW | TCAACTTCTCCAACGGCTCCAGCGTTTTCACCCTGAGCGCCCACGTGTTCAATTCCG |
| > GI54YUC01EFT5G | TCAACTTCTCCAACGGCTCCAGCGTTTTCACCCTGAGCGCCCACGTGTTCAATTCCG |
| > GI54YUC01EW3H0 | TCAACTTCTCCAACGGCTCCAGCGTTTTCACCCTGAGCGCCCACGTGTTCAATTCCG |
| > GI54YUC01CN62A | TCAACTTCTCCAACGGCTCCAGCGTTTTCACCCTGAGCGCCCACGTGTTCAATTCCG |
| > GI54YUC01E0L9O | TCAACTTCTCCAACGGCTCCAGCGTTTTCACCCTGAGCGCCCACGTGTTCAATTCCG |
| > GI54YUC01BF0OV | TCAACTTCTCCAACGGCTCCAGCGTTTTCACCCTGAGCGCCCACGTGTTCAATTCCG |
| > GI54YUC01EMAJY | TCAACTTCTCCAACGGCTCCAGCGTTTTCACCCTGAGCGCCCACGTGTTCAATTCCG |
| > GI54YUC01EW54M | TCAACTTCTCCAACGGCTCCAGCGTTTTCACCCTGAGCGCCCACGTGTTCAATTCCG |
| > GI54YUC01DZEEA | TCAACTTCTCCAACGGCTCCAGCGTTTTCACCCTGAGCGCCCACGTGTTCAATTCCG |
| > GI54YUC01AON4A | TCAACTTCTCCAACGGCTCCAGCGTTTTCACCCTGAGCGCCCACGTGTTCAATTCCG |
