# Supplementary material for: The Use of Massive Sequencing to Detect Differences between Immature Embryos of MON810 and a Comparable Non-GM Maize Variety
Source: PLoS One. 2014 Jun 26;9(6):e100895. doi: 10.1371/journal.pone.0100895 (PMC4072715; doi:10.1371/journal.pone.0100895)
Supplement: Table S7 — Oligonucleotides used in this study. Bold-underlined sequences are meant to directionally ligate 454 pyrosequencing adaptors. (DOCX) [file pone.0100895.s012.docx]

**Table S7.** Oligonucleotides used in this study. Bold-underlined sequences are meant to directionally ligate 454 pyrosequencing adaptors.

| **Name** | **454 library construction** | | **Use** |
| --- | --- | --- | --- |
| *Gsu-dT* | GAGCTCCTT*CTGGAG*AGTTTTTTTTTTTTTTTVN | | 454-cDNA library synthesis |
| A | CCATCTCATCCCTGCGTGTCTCCGACTCAG | | 454 A-adaptor 5’-3’ strand |
| A’ | **CG**CTGAGTCGGAGACACG | | 454 A-adaptor 3’-5’ strand |
| B | CTGAGACTGCCAAGGCACACAGGGGATAGG | | 454 B-adaptor 5’-3’ strand |
| B’ | Biotin-CCTATCCCCTGTGTGCCTTGGCAGTCTCAG**TT** | | 454 B-adaptor 3’-5’ strand |
| Zm-upl-F | CCCTTCTTGGAGAACCTCAGAGC | | *Zm-upl* expression analysis |
| Zm-upl-R | CCAAACTCAGATGAATCAAAAGGACCT | |  |
| Cyc-F | GCTCCCAGTTCTTCATCTGC | | RT-qPCR housekeeping |
| Cyc-R | CTAGGAGAGCTGTCCGCAGT | |  |
|  | **Upregulated sequences (5’- 3’)** | | **MaizeGDB accession** |
| Upreg1 | CACCCTTGATGTTGTTCGTG | CCATCCTGTGTCCTCTTGGT | GRMZM2G466833 |
| Upreg2 | GCGACAACATCAACACCATC | ACCCTCAAACGAGGGAAGTT | GRMZM2G027862 |
| Upreg3 | GCCATGGACAGAAACTGGAT | CACGACTGAGCAAACCAAGA | GRMZM2G134251 |
| Upreg4 | CGGCTGCTTCTACATTCACA | GTCCGAGTCATCCAACACCT | GRMZM2G028286 |
| Upreg5 | TACCCCAAGAGCCAGCTTTA | GCTGTATCCGGTCCTGTTGT | GRMZM2G447795 |
| Upreg6 | ATTCTGGACAGGATGCCAAC | TGCTGCCTTTGCAATAAGTG | GRMZM2G013811 |
| Upreg7 | GGATACAGTGAAGGGGCTCA | ACTTCTGCGCCAAGAAGGTA | GRMZM2G172369 |
| Upreg8 | CCAACATCCACACTGTGCTC | GCTGCAGGGACAAGAACATT | GRMZM2G028955 |
| Upreg9 | CGCTGTGTTCTGTGGTGTCT | AAGCAACAGCAGCCCTATGT | GRMZM2G421279 |
| Upreg10 | CGAACCAACATGTTTTCGTG | TATGCGACCACACCACCTTA | GRMZM2G072855 |
| Upreg11 | TCTGGCTATCCGTTCTTTGG | GCCAGACAGAGTGCAGTTCA | GRMZM2G181153 |
| Upreg12 | GCAGCGAGGGATAACAAGAA | TAGCGAATGCCTTCTCCAGT | GRMZM2G151826 |
| Upreg13 | TCGTTTCGGAGAAAACAAGC | TGGATTGGAGACGATGATGA | GRMZM2G093325 |
| Upreg14 | GCCAAGGCAGAGAAGAAGAA | AAGGTGAGGAGGAGGCAGAT | GRMZM2G015605 |
| Upreg15 | TCAAGCTCTCCATCCAGCTT | CTGCACGCGTACGTAAGAAA | GRMZM2G040095 |
|  | **Upregulated sequences (5’- 3’)** | | **MaizeGDB accession** |
| Dnreg1 | ACTGGAGGCCAAACACATTC | AAGAAGGGTCGGGGAGAGTA | GRMZM2G097229 |
| Dnreg2 | CTCTCCGCTTCCAAGATGTC | CCCTTGATTCCCTTCACAGA | GRMZM2G102230 |
| Dnreg3 | AGCTCCGCCACAAGTACAAC | TTGGTGACGATCACTTTGGA | GRMZM2G327564 |
| Dnreg4 | ACATGCCGGATTGAGAAAAG | GTAGCCCCACTCGTTGTCAT | GRMZM2G104632 |
| Dnreg5 | GAGCGCTGGGTAATTGGATA | GATCATCTTGCTCGGGAGAG | GRMZM2G118003 |
| Dnreg6 | ATCCGCAATTTAACCACTCG | GAAGAGGAGGAGGAGGAGGA | GRMZM2G167637 |
| Dnreg7 | GGACGAGAAGCTCAAGTTCG | GAACTCCTCCATGTCCTCCA | GRMZM2G140201 |
| Dnreg8 | GCCAAATTGGAGCGATAAAA | TCATCGAGCAAATTGACTGC | GRMZM2G174757 |
| Dnreg9 | CGAACTCAGGTTGGGTGATT | ATCTCTCAGCACGCCTGAAT | GRMZM2G100120 |
| Dnreg10 | AGCCAGAACGACAAGGAGAA | CTGTGCCTTCTTCCAAAAGC | GRMZM2G071333 |
| Dnreg11 | AGGGGGTTACACCTGAGCTT | TTCCCTTCATCCGATCTGTC | GRMZM2G046191 |
| Dnreg12 | GCTGTGGGAAGGAGCAGTAG | CTCACCACCAGGAGGAACAT | GRMZM2G083173 |
| Dnreg13 | CGTGTTCATAGCACCACCAC | TCCCAGCGTTACAGTGACAG | GRMZM2G018375 |
| Dnreg14 | GCCAGAACTGGACGAGTAGC | CAACACGCGTTATTGGACAC | GRMZM2G407044 |
| Dnreg15 | CAGTGCTGAAGCAATCCTGA | GTCCGGAAGAGGAAGGGTAG | GRMZM2G179981 |
