# Supplementary material for: The Use of Massive Sequencing to Detect Differences between Immature Embryos of MON810 and a Comparable Non-GM Maize Variety
Source: PLoS One. 2014 Jun 26;9(6):e100895. doi: 10.1371/journal.pone.0100895 (PMC4072715; doi:10.1371/journal.pone.0100895)
Supplement: Table S8 — Morphological measurements (mean ± S.E.) of dry weight, axis length and embryo area for the MON810 – near isogenic variety pairs DKC6575-Tietar, PR33P67-PR33P66 and DKC6041YG-DKC6040. Statistical analysis of means (t-test p-value, significance level of 0.05) for each parameter is show on the lower part of the table. (DOCX) [file pone.0100895.s013.docx]

|  | 20 DAP Embryos | | |
| --- | --- | --- | --- |
| Varieties | Dry weight (mg) | Axis length (cm) | Area (mm2) |
| DKC6575 | 56.65 ± 11.79 | 0.68 ± 0.06 | 69.80 ± 7.13 |
| Tietar | 59.48 ± 11.37 | 0.70 ± 0.10 | 74.04 ± 6.54 |
| PR33P67 | 56.15 ± 10.41 | 0.69 ± 0.06 | 63.15 ± 7.33 |
| PR33P66 | 59.20 ± 13.19 | 0.73 ± 0.09 | 63.07 ± 1.34 |
| DKC6041YG | 67.60 ± 9.67 | 0.64 ± 0.06 | 69.31 ± 9.33 |
| DKC6040 | 63.30 ± 7.96 | 0.65 ± 0.11 | 72.90 ± 7.62 |
|  |  |  |  |
| Statistical analysis |  |  |  |
| DKC6575 vs Tietar | 0.5915 (n.s.) | 0.5357 (n.s.) | 0.2159 (n.s.) |
| PR33P67 vs PR33P66 | 0.6577 (n.s.) | 0.4545 (n.s.) | 0.9965 (n.s.) |
| DKC6041YG vs DKC6040 | 0.5181 (n.s.) | 0.9230 (n.s.) | 0.4927 (n.s.) |

Table S8. Morphological measurements (mean ± S.E.) of dry weight, axis length and embryo area for the MON810 – near isogenic variety pairs DKC6575-Tietar, PR33P67-PR33P66 and DKC6041YG-DKC6040. Statistical analysis of means (t-test p-value, significance level of 0.05) for each parameter is show on the lower part of the table.
